# Devastating Rio Doce mining disaster sends shockwaves through earthworm populations

**DOI:** 10.1002/jeq2.70056

**Published:** 2025-07-01

**Authors:** Herlon Nadolny, Yumi Oki, Walisson Kenedy‐Siqueira, Marcos P. Santos, Luis M. Hernández‐García, Daniel Negreiros, João C. G. Figueiredo, Fernando F. Goulart, George G. Brown, Geraldo W. Fernandes

**Affiliations:** ^1^ Instituto de Ciências Biológicas Universidade Federal de Minas Gerais Belo Horizonte Minas Gerais Brazil; ^2^ Knowledge Center for Biodiversity Belo Horizonte Minas Gerais Brazil; ^3^ Centro de Ciências Agrárias Universidade Estadual do Maranhão São Luís Maranhão Brazil; ^4^ Departamento de Biologia Geral Universidade Estadual de Montes Claros Montes Claros Minas Gerais Brazil; ^5^ Embrapa Florestas Curitiba Paraná Brazil

## Abstract

The Fundão dam breach is considered one of the most severe environmental mining disasters globally, causing widespread changes to the soils of the Rio Doce watershed, one of Brazil's most important catchments. Given the ecological importance of earthworms to soil structure and dynamics, we investigated the richness, abundance, and biomass of both native and invasive earthworm species in riparian zones along the Rio Doce to understand their responses to the altered soil conditions. Sampling was conducted in reference and impacted sites across five municipalities in Minas Gerais: Mariana, Rio Casca, Ipatinga, Conselheiro Pena, and Aimorés. We identified eight species—two invasive (*Amynthas gracilis* and *Pontoscolex corethrurus*) and six native (two *Rhinodrilus*, three *Righiodrilus*, including at least two undescribed species, and one *Ocnerodrilidae* species)—with native biomass approximately five times lower in impacted sites compared to reference sites. Furthermore, the new tailings environment altered the relationships between soil properties and earthworm abundance for both native and invasive species. These findings indicate that native earthworms are less tolerant of the disturbed soil conditions than invasive species, which may contribute to shifts in community composition. The disruption of soil‐fauna interactions underscores the long‐term ecological consequences of mining‐related disturbances and highlights the need for restoration efforts that consider belowground biodiversity.

## Core Ideas

- Earthworms are important soil contamination indicators.
- Rio Doce watershed is home to a unique earthworm richness, where two new native species were recorded.
- Tailings from the Fundão dam collapse changed the soil's physicochemical properties, impacting earthworms.
- The distribution of earthworm species is strongly influenced by specific soil properties.
- Native earthworms are more sensitive to soils impacted by tailings deposition compared to invasive earthworms.

**Abbreviations**
Bsat: base saturation
COIA: co‐inertia analysis
CoS: coarse sand
eCEC: effective cation exchange capacity
FiS: fine sand
GLMM: generalized linear mixed models
IS: impacted sites
PCA: principal components analysis
PDens: particle density
RS: reference sites
SOM: soil organic matter

## 1 INTRODUCTION

Soil is a complex and vital component for life on Earth and contains approximately 23% of all described animal species (Anthony et al., 2023; Decaens et al., 2006). Among these organisms, earthworms make up more than 80% of the soil fauna biomass and act as ecosystem engineers, changing the soil physically as a habitat and affecting the availability of resources for other organisms (Jones et al., 1994; Lavelle et al., 2016). They create air and water circulation channels, play a role in nutrient cycling, and decompose soil organic matter (SOM) through microbial activity priming (Lavelle, 1988). The importance of earthworms in shaping ecosystem dynamics is well‐documented (e.g., Blouin et al., 2013; Ruiz et al., 2021). However, only one comprehensive review on their distribution, diversity, and influencing factors has been published to date (Phillips et al., 2019), though its findings have been contested (James et al., 2021).

Over 6000 earthworm species have been described (Csuzdi, 2012), though only around 5753 are valid (Brown et al., 2024; Misirlioğlu et al., 2023). In Brazil, nearly 340 earthworm species have been identified (Brown et al., 2013), while 1400 species were estimated to inhabit the country (James & Brown, 2006). This highlights a significant knowledge gap regarding earthworm distribution, diversity, and natural history in the country. Their ecology, including relationships with soil and plants, remains largely unexplored, particularly for native species (Brown et al., 2013).

In general, earthworm richness, abundance, and biomass are affected by climate, as well as vegetation cover and various soil properties, such as pH, texture, and nutrient contents (e.g., Edwards, 2004; Fragoso & Lavelle, 1992; Phillips et al., 2019; Rutgers et al., 2016, 2019). Soils with extreme pH values (acidic or alkaline) and high concentration of clay or sand affect native earthworm survival and performance (Edwards & Arancon, 2022; S. Singh et al., 2020; Tamartash & Ehsani, 2021). However, little is known regarding the chemical or physical soil preferences of most earthworm species, particularly in the tropics (Brown et al., 2013). Tropical litter‐dwelling earthworm species are typically found in oligotrophic soils, whereas soil‐dwelling species are more prevalent in eutrophic soils (Fragoso & Lavelle, 1992). However, there is limited published research on the relationships between earthworm communities (in terms of abundance and richness) and soil properties in Brazilian forests. Most of the available information is limited to unpublished graduate theses or dissertations (e.g., Beckman, 2019; Hernández‐García, 2019; Lima, 2011; Patucci, 2015; Pereira, 2012). Because of their sensitivity to soil parameters, and their relative ease of collecting and laboratory culture (Alves & Cardoso, 2016; Helling et al., 2000; Lukkari et al., 2005), earthworms are frequently used as bioindicators of ecosystem disturbance (Paoletti, 1999), soil health (Bünemann et al., 2018), and the level of contamination by heavy metals or pesticides (Pelosi et al., 2014; Yadav et al., 2023).

The most widespread and well‐known earthworm species are typically those with broad ecological niches, and they are generally the ones most resistant to the impacts of land use change and soil management practices (Taheri et al., 2018). These anthropochory species (Gates, 1972) are peregrines found worldwide, and include over 100 species, many of them invasives (Hendrix et al., 2008). Moreover, these invasive earthworms can alter soil quality, drive out native earthworms through competition, and reduce populations of soil macrofauna (Demétrio et al., 2023; Zhang et al., 2010). All these changes can pose a serious threat to ecosystem functioning and biodiversity (Ferlian et al., 2017).

The catastrophic event triggered by the rupture of the Samarco's Fundão dam in 2015 in Mariana led to the discharge of dozens of millions of cubic meters of iron ore tailings into the Rio Doce watershed, one of Brazil's crucial river systems (Carmo et al., 2017; G. W. Fernandes et al., 2016; Fonseca & Fonseca, 2016). The mining tailings traveled approximately 650 km before reaching the Atlantic Ocean destroying much of the aquatic and riparian biota along the way (G. W. Fernandes et al., 2016; Miranda & Marques, 2016; A. C. O. Neves et al., 2016; Quadra et al., 2019). Furthermore, the impact resulted in significant alterations to the soil structure along extensive riparian zones, causing a drastic modification of the local topography, and leading to the formation of a widespread deposit of nutrient‐poor material with high silt content and substantial concentrations of iron (Fe), aluminum (Al), arsenic (As), chromium (Cr), cadmium (Cd), mercury (Hg), manganese (Mn), and lead (Pb) (Hatje et al., 2017; Figures S1 and S2). The severity of the disaster shows that the particulate material was not restricted to the river channel, but was widely distributed across the landscape (Carmo et al., 2017; G. W. Fernandes et al., 2016; Gabriel et al., 2021; D. S. Neves et al., 2024). The profound physical and chemical changes in the soil following this disaster caused drastic reductions in the diversity of plants (G. W. Fernandes et al., 2025) and microorganisms (Giongo et al., 2020; Silva et al., 2021) in the directly affected riparian sites. However, there is no information on whether these changes in the ecosystem affected the diversity and biomass of ecosystem engineers, including earthworms. Given their ecological importance and sensitivity to soil disturbances, earthworms were therefore selected as key indicators to assess potential soil contamination and to support decision‐making processes related to the mitigation of this environmental disaster, as well as to inform monitoring and restoration programs at the affected sites. We tested the following hypotheses: (1) earthworm richness, abundance, and biomass are lower in sites impacted by the tailings (impacted sites [IS]) compared to reference sites (RS) (no direct contact with mine tailings). The RS followed the criteria described in Toma et al. (2023), that is, best preserved sites that serve as a model to guide environmental restoration (for site details, see Figueiredo et al. [2022, 2024], Ramos et al. [2023, 2024], and G. W. Fernandes et al. [2025]); (2) earthworm species are influenced by soil properties. Earthworms are sensitive to variations in soil physicochemical properties, as these characteristics directly or indirectly influence the availability of resources for their survival (S. Singh et al., 2016); (3) changes in soil properties and vegetation cover in the IS decrease native earthworm species abundance and biomass; and (4) invasive earthworm species present higher biomass in IS compared to RS, since they are highly tolerant of habitat disturbance and adverse conditions.

## 2 MATERIALS AND METHODS

### 2.1 Study areas

**FIGURE 1:**
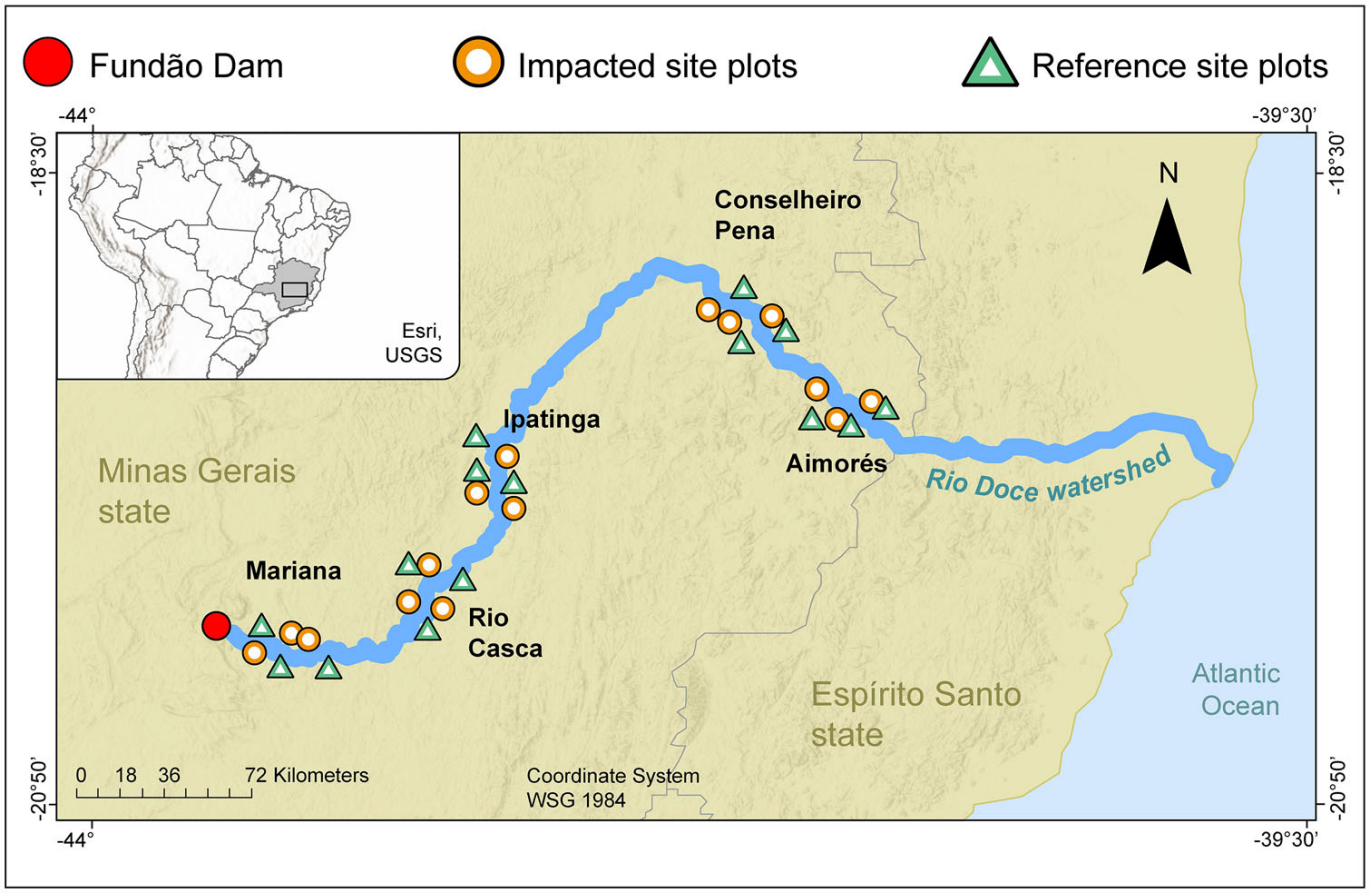
Map showing the location of the 30 sampling sites (green triangles = reference sites; orange circles = impacted sites) in the five main regions along the Rio Doce watershed, Minas Gerais state, Brazil.

The study was carried out in five regions located along the Rio Doce watershed (Figure 1; Table S1) in Minas Gerais State, southeast Brazil: Mariana, Rio Casca, Ipatinga, Conselheiro Pena, Aimorés. The Rio Doce watershed encompasses two biodiversity hotspots (Mittermeier et al., 2004): Atlantic Rainforest (98% of its area) and Cerrado (2% of its area) (Ribeiro et al., 2020). The Atlantic Forest vegetation found in the five regions sampled is predominantly semi‐deciduous seasonal forests (G. W. Fernandes et al., 2025; Ramos et al., 2024). In each of the five regions, six sampling sites—three IS and three RS—were selected, each spaced 2–3 km apart, totaling 30 sites (*n* = 30), to evaluate the earthworm community (Figures S1 and S3). The selection of the reference and impacted areas was based on remote sensing imagery and technical field visits initiated in March 2017. At that time, the contrast between reference and impacted areas was clear. In the impacted areas, visible marks of tailings flow could be seen on tree trunks, along with significant changes in the texture of the surface soil.

**Core Ideas**

- Earthworms are important soil contamination indicators.
- Rio Doce watershed is home to a unique earthworm richness, where two new native species were recorded.
- Tailings from the Fundão dam collapse changed the soil's physicochemical properties, impacting earthworms.
- The distribution of earthworm species is strongly influenced by specific soil properties.
- Native earthworms are more sensitive to soils impacted by tailings deposition compared to invasive earthworms.

The climate of the sampled sites is Cwa and Aw according to the Köppen classification (Alvares et al., 2013) (Table S1). The Cwa climate type is characterized by a humid subtropical climate with hot, humid summers and dry winters, while Aw has extremely dry winters and low precipitation (Alvares et al., 2013). Mariana has higher rainfall and lower minimum and maximum temperatures compared to Aimorés (Figure S4; Table S2, Climatem).

Fifteen plots measuring 10 m × 10 m (100 m^2^) were established in each sample site (= 1500 m^2^). This sampling effort was triplicated for each condition, that is 45 plots each for IS and RS, for a total of 90 plots (9000 m^2^) (Figure S3). For all five sampled regions we established 225 plots in RS (22,500 m^2^) and another 225 plots (22,500 m^2^) in IS. Plots were separated from each other by 10 m (*n*_total_ = 44,500 m^2^).

### 2.2 Soil properties

Soils of all sites are derived from alluvial deposits of the Rio Doce watershed and constitute mainly recent soil types (Entisols: USDA classification, Soil Survey Staff, 2010) with coarser to loamy texture (higher sand contents) or well‐developed older soils with more loamy or finer texture (higher silt and clay contents) (Guevara et al., 2018; Ramos et al., 2024). The soil surface is generally acidic, with low P availability, low cation exchange capacity, and SOM exceeding 2.5% on average (Guevara et al., 2018).

In general, the IS in all the locations studied stood out for having low concentration of C (average proportion C_IS _= 1.2%; C_RS _= 1.7%) and a high concentration of Fe and P (Table S3 for more details). The Fe (average concentration Fe_IS _= 144.9 mg/dm^3^) and P (average concentration P_IS _= 6.6 mg/dm^3^) concentrations were twice as high in the IS as in the RS. In terms of soil texture, the IS had a higher proportion of fine sand (FiS; average proportion = 38.5%) and a lower proportion of coarse sand (CoS; average proportion = 17.7%) than the RS (average proportion of FiS = 25.4%; average proportion of CoS = 30.2%).

### 2.3 Earthworm sampling and identification

Earthworms were collected 7 years after the Fundão dam collapse (November 5, 2015), during the rainy season of March 2023. The rainy period is considered the most suitable time to sample these organisms, since the soils have adequate moisture levels (Nadolny et al., 2020). An adaptation of the *Tropical soil biology and fertility* method (Anderson & Ingram, 1993) was used to sample the earthworm community. In each plot we excavated a single soil monolith measuring 0.2 m × 0.2 m on a side (evenly spaced), to a depth of 0.2 m, using a straight or square shovel (*n* = 15 plots per site × 6 sites = 90 plots per region × 5 regions = 450 plots). Earthworm samples were distanced at least 20 m from each other. The soil from each monolith was placed in a plastic tray and gently broken up to remove the earthworms, which were placed in labeled plastic jars containing 90% alcohol. The number of individuals (abundance) per monolith was counted. Subsequently, the number of individuals per square meter was calculated. The material was stored in a refrigerator (−4°C) and the absolute alcohol in the vials was changed after arriving from the field, and then at every 24 h for 3 consecutive days. The alcohol material does not freeze at −4°C and helps to preserve the material until it reaches the laboratory, preserving structures if necessary to be used for molecular analysis.

Earthworms were initially separated into morphospecies, and all the individuals (ind) were weighed separately by morphospecies and development stage (adult or juvenile) using an analytical balance (0.01 g). Taxonomic identification (juveniles and adults) was performed using the keys of Righi (1995), Blakemore (2002), and Hernández‐García et al. (2018). Some specimens could only be identified to the genus or family level. After taxonomic identification, the species were classified as native or invasive according to Demetrio et al. (2023).

### 2.4 Native forest cover

Native forest cover at each sampling site was assessed using Landsat8 satellite imagery from the study period. We used 1 km buffers around the sampling sites and overlaid these with vegetation maps developed by MapBiomas (https://mapbiomas.org/) at 30 m × 30 m resolution. The analysis was carried out at the largest and closest scale (1000 m). Although each buffer always has a radius of 1000 m, the number of pixels varied, so the area referred to in this study was equal to the number of pixels multiplied by 900 m^2^, as each pixel was 30 m × 30 m. The landscape‐scale native forest cover of the IS and RS along the river was examined to assess whether the proportion of this parameter around the sites affected earthworm biomass.

### 2.5 Statistical analyses

We performed co‐inertia analysis (COIA) to evaluate the relationship between soil attributes and earthworm species using the “ade4” package (Dray & Dufour, 2007; Dray et al., 2003). The soil matrix included 18 soil parameters from 98 plots, while the community matrix included incidence data of eight earthworm species. The strength of the association was assessed using the RV coefficient, with significance determined via Monte Carlo permutation (10,000 randomizations). Principal components analysis (PCA) was used for the soil matrix and centered PCA for the earthworm community matrix. Furthermore, we used Pearson's correlation to assess the association between each soil parameter and COIA axis 1, while we used species coordinates to determine the association with COIA axis 1.

Generalized linear mixed models (GLMM) were constructed to investigate the effects of native forest cover and soil attributes on earthworm total abundance, abundance of native and invasive earthworms, biomass of total earthworm, and biomass of native and invasive earthworms found in IS and RS. The interactions between IS and RS and environmental variables were used as explanatory variables. Sites and regions were considered as random effects to be controlled in each model. The models underwent analysis of variance testing and were deemed significant when *p*‐values were below 0.05. The “lme4” package (Bates et al., 2015) was used to conduct these analyses in the R environment (R Core Team, 2023).

The *R* ^2^ values represent a pseudo‐*R* ^2^ value that describes the fit of the model to the data. The *R* ^2^ marginal is the variance explained by the fixed effects, while the *R* ^2^ conditional is the variance explained by the fixed and random effects. The *R* ^2^ values of all models were calculated using the Performance package (Lüdecke et al., 2021).

## 3 RESULTS

### 3.1 Local and regional earthworm species richness

Of the total of eight species sampled in this study, two species are non‐native and invasive in the Atlantic Forest biome: *Amynthas gracilis* (Megascolecidae, originally from Asia) and *Pontoscolex corethrurus* (Rhinodrilidae, Origin: Guyana shield) (Demetrio et al., 2023). The other earthworm species/morphospecies were *Rhinodrilus motucu*, *Rhinodrilus* sp.1 (Rhinodrilidae), *Righiodrilus* sp.1, *Righiodrilus* sp.2, and *Righiodrilus* sp.3 (Glossoscolecidae), and juveniles of an *Ocnerodrilidae* sp. At least two species are new to science (*Righiodrilus* sp.2, and *Righiodrilus* sp.3). These two new species show variations in certain organs that can hardly be attributed to a single mutation. These changes affect the position of reproductive structures, which significantly reduce the likelihood of gene exchange with other species (Pavlíček et al., 2012).

Six earthworm species were recorded in the IS: the two invasive species, *Rhinodrilus* sp.1, *Righiodrilus* sp.1, n.sp.2, and *Ocnerodrilidae* sp. In the RS, we recorded seven out of the eight earthworm species, of which five were native (*R. motucu*, *Rhinodrilus* sp. 1, *Righiodrilus* sp.1, n.sp.2, and n.sp.3) and the two invasive species. Regional earthworm species richness ranged from a minimum of one (Aimorés, IS) up to four (Mariana, IS; Table 1). On the other hand, earthworm species richness per plot did not differ statistically between IS (average ± standard error = 1.06 ± 0.005) and RS (1.08 ± 0.006) (*p* > 0.05).

**TABLE 1:** Earthworm species recorded in sites impacted by mining tailings (225 sampling plots) and in reference sites (225 sampling plots) distributed along the riparian zone of the Rio Doce watershed in five regions (Mariana, Rio Casca, Ipatinga, Conselheiro Pena, and Aimorés) of Minas Gerais state, Brazil.

Earthworm	Mariana		Rio Casca		Ipatinga		Conselheiro Pena		Aimorés		Total (IS/RS)
	IS	RS	IS	RS	IS	RS	IS	RS	IS	RS	
*Pontoscolex corethrurus* (Rhinodrilidae)^a^	104	160	28	64	75	48	68	3	22	7	579 (297/282)
*Amynthas gracilis* (Megascolecidae)^a^	1	2	1	0	2	1	1	0	0	2	10 (5/5)
*Rhinodrilus motucu* (Glossoscolecidae)	0	0	0	5	0	0	0	0	0	0	5 (0/5)
*Rhinodrilus* sp.1 (Glossoscolecidae)	0	0	0	0	0	0	4	9	0	1	14 (4/10)
*Righiodrilus* sp.1 (Glossoscolecidae)	1	0	2	0	0	0	0	1	0	0	4 (3/1)
*Righiodrilus* sp.2 (Glossoscolecidae)	3	2	0	0	0	0	0	0	0	0	5 (3/2)
*Righiodrilus* sp.3 (Glossoscolecidae)	0	0	0	0	0	1	0	0	0	0	1 (0/1)
Ocnerodrilidae sp. juveniles (Ocnerodrilidae)	0	0	0	0	2	0	0	0	0	0	2 (2/0)
Total	109	164	31	69	79	50	73	13	22	10	620 (314/306)
Regional species richness (*n* species)	4	3	3	2	3	3	3	3	1	3	8 (6/7)
Mean species richness (*n*/site)	1.3	1	1	0.7	1	1	1	1	0.3	1	(0.4/0.47)
Mean species richness (*n*/plot)	0.27	0.2	0.2	0.13	0.2	0.2	0.2	0.2	0.07	0.2	(0.027/0.031)

^a^ Invasive earthworm species.

Abbreviations: IS, impacted sites; RS, reference sites.

### 3.2 Abundance and biomass of native and invasive earthworms

A total of 620 earthworm individuals were recorded, of which 95% (589 ind) belong to the two invasive species, and only 5.0% (31 ind) were of species native to the Atlantic Forest (Table 1). The total number of invasive earthworms was higher in IS than in RS (302 and 287 ind, respectively), while the number of native earthworms was lower in IS compared to RS (12 vs. 19 ind, respectively). The invasive *P. corethrurus* was recorded in all sites, independently of IS or RS, and represented 94.5% and 92% of all individuals collected in IS and RS plots, respectively. On the other hand, the presence of native species was highly variable (Table 1; Tables S4 and S5), with all specimens collected being juveniles (data not shown), and identifiable only to genera.

The abundance recorded in each region was highly variable, with most of the specimens being found in Mariana (273 ind, 44% of total), where soil moisture conditions were less drastic at sampling compared with the remaining regions, particularly Aimorés, where rainfall was lower (Table S2) and the soil drier during sampling (H. Nadolny, personal observation). Nonetheless, some of the sites in both Rio Casca and Conselheiro Pena also had dry soils during the sampling period (H. Nadolny, personal observation).

Mean of total earthworm (all species together) abundance (Table S4) and biomass (Table S5) per sampling plot did not differ statistically between IS (average ± standard error = 1879.0 ± 198.0 ind/m^2^ and 20.1 ± 2.8 g/m^2^, respectively) and RS (1649 ± 214 ind/m^2^ and 21.3 ± 2.3 g/m^2^, respectively) plots (total abundance: *χ* ^2 ^= 1.39, *p* = 0.24; total biomass: *F* = 0.0093, *p* = 0.92). Furthermore, the abundance and invasive earthworm biomass in IS (94.4 ± 8.4 ind/m^2^ and 20.8 ± 3.0 g/m^2^, respectively) was also not statistically different to that in RS (94.4 ± 9.71 ind/m^2^ and 18.4 ± 2.2 g/m^2^) plots (invasive earthworm abundance: *χ* ^2 ^= 0.29, *p* = 0.59; invasive earthworm biomass: *F* = 0.40, *p* = 0.53). Conversely, although mean of native earthworm abundance was not different between IS and RS (*χ* ^2 ^= 1.96; *p* = 0.16), mean biomass was significantly lower in IS than RS plots (*F* = 5.04, *p* = 0.048; Tables S6 and S7). Mean of native earthworm biomass was almost five times lower in IS (5.5 ± 3.0 g/m^2^) than in RS (28.1 ± 6.4 g/m^2^) (Figure S5).

### 3.3 Overall association between earthworm species and soil properties

**FIGURE 2:**
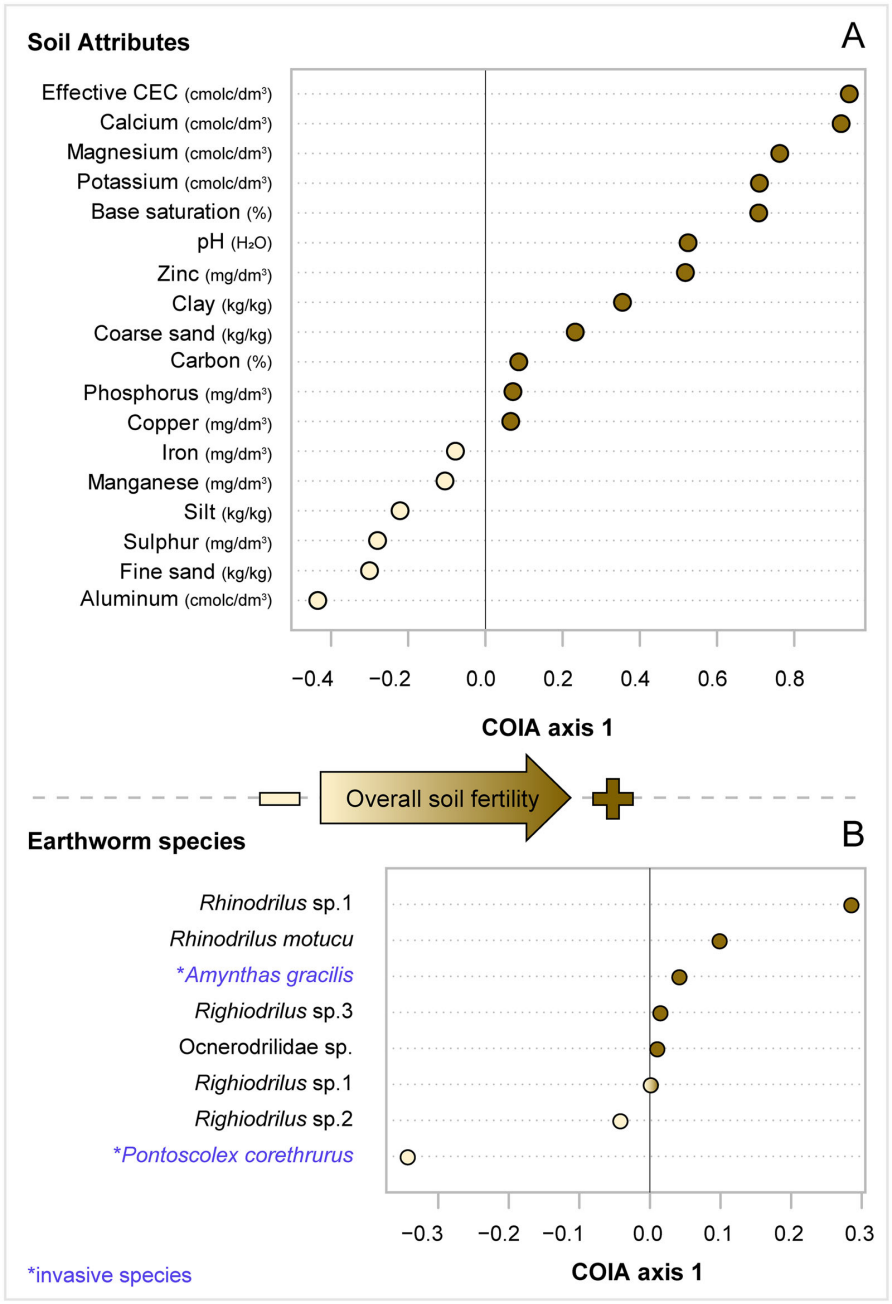
Co‐structure between edaphic attributes and earthworm community sampled along the Rio Doce watershed, southeast Brazil. The upper dotchart (A) shows the Pearson correlation between edaphic attributes and the coordinates of the sites in axis 1 of the co‐inertia analysis (COIA). The bottom dotchart (B) shows the coordinates of earthworm species in the COIA axis 1. The light brown and dark brown circles represent, respectively, the negative and positive correlation values with COIA axis 1 (upper graphs) or coordinates of COIA axis 1 (bottom graphs).

We observed a clear earthworm taxon–environment relationship, since the overall association between species and edaphic attributes was highly significant (RV coefficient = 0.194; *p* < 0.001). We found an association of 19.4% between the edaphic and community matrices. The percentage of covariance explained by axis 1 was 93.6% and for this reason we only explore the COIA axis 1. The positive side of COIA axis 1 showed plots with less acidic and more fertile soils, with higher effective cation exchange capacity (eCEC), Ca, Mn, K, base saturation (Bsat), Zn, and clay (Figure 2A). The earthworm species most strongly associated with the positive side of this axis were *Rhinodrilus* sp.1, and *R. motucu* (Figure 2B). On the other hand, the negative side of the COIA axis 1 showed plots with less fertile and more acidic soils, with higher Al, FiS, S, and silt (Figure 2A). The earthworm species most strongly associated with the negative side of this axis was the invasive *P. corethrurus* (Figure 2B).

### 3.4 Effect of native forest cover on total, native, and invasive earthworms

**FIGURE 3:**
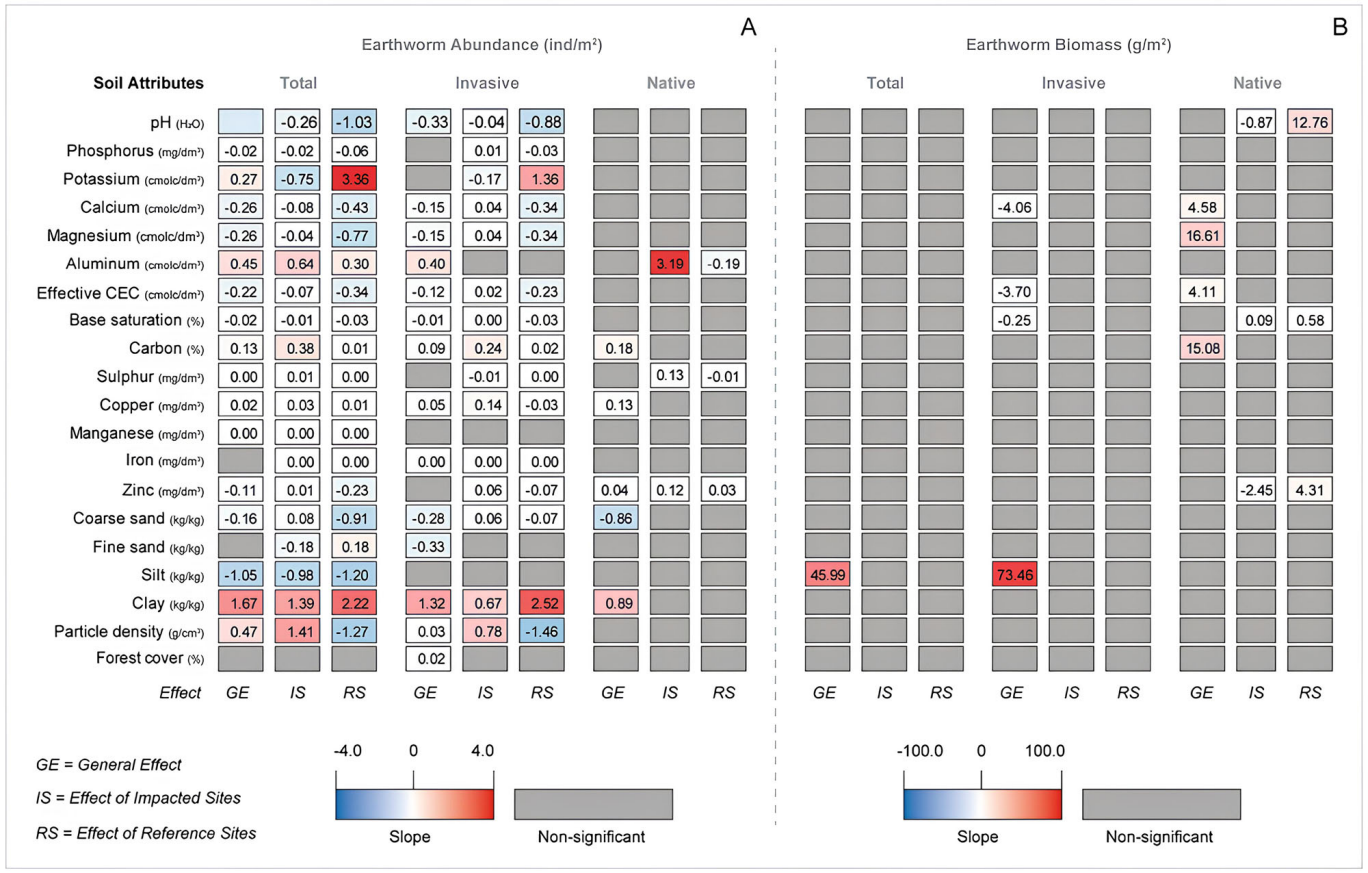
Slope results of generalized linear mixed models (GLMM) constructed to investigate the general effect (GE) of each soil attribute and forest cover (%) on (1) earthworm total abundance (ind/m^2^), (2) invasive earthworm abundance (ind/m^2^), (3) native earthworm abundance (ind/m^2^), (4) total earthworm biomass (g/m^2^), (5) invasive earthworm biomass (g/m^2^), and (6) native earthworm biomass (g/m^2^). The interaction between impacted sites (IS) and reference sites (RS) and environmental variables were used as explanatory variables.

The GLMM results indicated that the native forest cover (%) did not influence the abundance of total earthworms (*p* = 0.06) and of native earthworms (*p* = 0.51) in the riparian zones of the Rio Doce watershed (Tables S6 and S7). On the other hand, the percentage of native forest positively influenced the invasive earthworm abundance (*p* = 0.047; Figure 3; Tables S6 and S7). Each 1% increase in native forest cover led to an increase of 0.016 invasive earthworms. Even when environmental disaster conditions (IS vs RS plots) were included in the GLMM model, the native forest cover (%) did not influence the abundance of all earthworms, invasive earthworms, and native earthworms (*p* > 0.05; Tables S6 and S7).

The native forest cover (%) did not influence the total earthworm biomass (*p* = 0.81), invasive earthworm biomass (*p* = 0.36), or native earthworm biomass (*p* = 0.42) (Tables S6 and S7). The native forest cover (%) in the IS or RS plots had no influence on total earthworm biomass (*p* = 0.46), invasive earthworm biomass (*p* = 0.24), and native earthworm biomass (*p *= 0.45) (Tables S6 and S7).

### 3.5 Effect of soil properties and site conditions on total, invasive, and native earthworm abundance

#### 3.5.1 Soil properties on earthworm total abundance

Seventeen physical and chemical soil properties (pH, P, K, Ca, Mg, Al, eCEC, Bsat, C, S, Cu, Mn, Zn, CoS, silt and clay, and particle density [PDens]) correlated with earthworm total abundance (*p* < 0.05; Figure 3; Tables S6 and S7). The GLMM results indicated that most of the significant correlations between 11 soil properties (pH, P, Ca, Mg, eCEC, Bsat, S, Mn, Zn, CoS, and silt) and total soil abundance were negative (negative slopes, *p* < 0.001; Figure 3; Tables S6 and S7). In other words, each increase in the values of these 11 soil properties resulted in a decrease in the earthworm total abundance. Silt was the soil property that most negatively influenced the loss of earthworm total abundance (Figure 3). Each unit increase in silt content led to a decrease of one earthworm. On the other hand, the correlations between six other soil properties, K, Al, C, Cu, clay, and the soil PDens, and earthworm total abundance were positive (*p* < 0.001; Figure 3; Tables S6 and S7). Each increase in the concentration of K, P, Al, C, Cu, clay, and soil PDens led to an increase in the earthworm total abundance. Clay had the highest positive slope value in relation to earthworm total abundance (Figure 3). Each unit increase in clay content led to an increase of almost two earthworms.

#### 3.5.2 Soil properties and site conditions on earthworm total abundance

When we included the impacted or non‐impacted condition (IS vs. RS plots) in the model, the results indicated that the correlations between all soil properties and earthworm total abundance varied significantly between IS and RS (GLMM, *p* < 0.001; Figure 3; Tables S6 and S7). Seven soil properties, pH, P, Ca, Mg, eCEC, Bsat, and silt, negatively influenced earthworm total abundance in both IS and RS (*p* < 0.001). Each increase in the values of these seven soil properties led to a decrease in the earthworm total abundance, which was lower in IS. On the other hand, Al, C, and clay positively influenced earthworm total abundance (GLMM, *p* < 0.001; Figure 3; Tables S6 and S7). For each unit increase in Al and C content in soils, the earthworm total abundance in IS increased two and 47.5 times, respectively, compared to RS. For every unit increase in the content of clay in the soil, the earthworm total abundance in IS increased 1.6 times less than in RS. Finally, the relationship between the soil properties, K, Mn, and FiS, and the earthworm total abundance was negative in IS and positive in RS (*p* < 0.001; Figure 3; Tables S6 and S7). Each increase in the values of K, Mn, and FiS resulted in a reduction in the earthworm total abundance in IS and an increase in the earthworm total abundance in RS, while the relationship between the parameters S, Fe, Zn, and CoS was positive in IS and negative in RS (*p* < 0.001). Each increase in the values of S, Fe, and Zn led to an increase in the earthworm total abundance in IS and a reduction in this abundance in RS.

#### 3.5.3 Soil properties on invasive earthworm abundance

The relationship between eight soil properties (pH, Ca, Mg, Al, eCEC, Bsat, CoS, and FiS) and invasive earthworm abundance was negative (*p* < 0.001; Figure 3; Tables S6 and S7). In other words, the increase in the values of these eight soil properties resulted in a decrease in the invasive earthworm abundance. On the other hand, the relationship between soil properties, Al, C, Cu, Fe, clay, and soil PDens, and invasive earthworm abundance was positive. The increase in the values of P, C, Cu, Fe, clay, and soil PDens resulted in an increase in the invasive earthworm abundance. Clay stood out as having the highest positive slope value in relation to invasive earthworm abundance (Figure 3). The increase of each clay content unit contributed to the increase of one individual invasive earthworm.

#### 3.5.4 Soil properties and site conditions on invasive earthworm abundance

When we included site conditions (IS or RS) in the model, we observed that the relationship between the properties, pH and Bsat, and the invasive earthworm abundance was negative in both IS and RS. However, for each increase in pH and Bsat values resulted in a higher decrease in the invasive earthworm abundance in IS than in RS (*p* < 0.001; Figure 3; Tables S6 and S7). The relationship between the soil properties, C and clay, and invasive earthworm abundance was positive, although they differed statistically between IS and RS (*p* < 0.001; Figure 3; Tables S6 and S7). Each increase in C content led to a higher increase in the invasive earthworm abundance in IS than in RS (*p* < 0.001; Figure 3; Tables S6 and S7). The magnitude of the effect of C on the invasive earthworm abundance in IS was almost 11 times higher than in RS. While the increase in clay content values resulted in a lower increase in the invasive earthworm abundance in IS than in RS (*p* < 0.001; Figure 3; Tables S6 and S7), the magnitude of the clay's effect on the invasive earthworm abundance in IS was almost four times lower than in RS. We also noted that the relationship between soil properties, P, Ca, Mg, eCEC, C, Cu, Fe, Zn, and PDens, and invasive earthworm abundance was positive in IS and negative in RS (*p* < 0.001; Figure 3; Tables S6 and S7), while the relationship between the soil properties, K and S, and the invasive earthworm abundance was negative in IS and positive in RS (*p* < 0.001; Figure 3; Tables S6 and S7).

#### 3.5.5 Soil properties on native earthworm abundance

A positive relationship was found between the abundance of native earthworms and four soil properties: carbon (C), copper (Cu), zinc (Zn), and clay content (*p* < 0.05; Figure 3; Tables S6 and S7). Each increase in the concentrations of C, Cu, Zn, and clay resulted in higher native earthworm abundance. Clay stood out as the property with the highest positive slope value in relation to the native earthworm abundance (Figure 3). Each unit increase in clay content interferes with the increase of almost one individual native earthworm. CoS was the unique soil property in which an increase in concentration led to a decrease in the native earthworm abundance (*p* = 0.01; Figure 3; Tables S6 and S7). Each unit increase in CoS content interferes with the decrease of almost one individual native earthworm.

#### 3.5.6 Soil properties and site conditions on native earthworm abundance

Only three soil properties (Al, S, and Zn) showed a significant relationship with the native earthworm abundance considering the condition of the site (IS or RS). The relationship between soil properties, Al and S, and the native earthworm abundance was statistically positive in IS and negative in RS (*p* < 0.05; Figure 3; Tables S6 and S7). The relationship between Zn content and native earthworm abundance was positive in both IS and RS, but this relationship differed statistically between IS and RS (*p* = 0.02; Figure 3; Tables S6 and S7). Each unit increase in Zn content resulted in an almost five‐fold increase in the native earthworm abundance in IS compared to RS.

### 3.6 Effect of soil properties on total, invasive, and native earthworm biomass

#### 3.6.1 Soil properties and site conditions on total earthworm biomass

None of the soil properties evaluated influenced total earthworm biomass (*p* > 0.05; Tables S6 and S7), except for silt (*p* = 0.01; Figure 3; Tables S6 and S7). Every 1 unit increase in silt resulted in an increase of 46 g/m^2^ in earthworm biomass. However, when site condition was considered as part of the model, soil properties had no impact on earthworm biomass in either IS or RS (*p* > 0.05).

#### 3.6.2 Soil properties and site conditions on biomass of invasive earthworms

Biomass of invasive earthworms was influenced positively by silt (*p* < 0.01) and negatively by Ca (*p* = 0.018), eCEC (*p* = 0.006), and Bsat (*p* = 0.036; Figure 3; Tables S6 and S7). Each unit of silt increase resulted in an increase in the invasive earthworm biomass of 73.5 g/m^2^, while, for each unit of Ca, eCEC, and Bsat increase resulted in a decrease in the invasive earthworm biomass of 4.1, 3.7, and 0.3 g/m^2^, respectively. When site conditions were taken into account in the model, no effect of soil properties on the invasive earthworm biomass was found (*p* > 0.05).

#### 3.6.3 Soil properties and site conditions on native earthworm biomass

For native earthworms, Ca (*p* = 0.043), Mg (*p* = 0.01), eCEC (*p* = 0.027), and C (*p *= 0.01) positively influenced their biomass (Figure 3; Tables S6 and S7). Each increase of one unit of Ca, Mg, eCEC, and C led to an increase in the native earthworm biomass of 4.6, 16.6, 4.1, and 15.1, respectively. These results highlight Mg and C as the two soil properties that contributed most to the weight of native earthworm biomass. When site condition was included in the model, pH (*p* = 0.04), Bsat (*p* = 0.03), and Zn (*p* = 0.02) affected the native earthworm biomass differently between IS and RS. The increase in these three parameters (pH, Bsat, and Zn) led to a greater increase in the biomass of native earthworms in the reference areas compared to the impacted area. Each increase of one pH unit resulted in a decrease of 0.9 g/m^2^ of native earthworm biomass in IS and an increase of 12.8 g/m^2^ of native earthworms in RS. Each increase of one Bsat unit resulted in an increase in native earthworm biomass of 0.1 g/m^2^ in IS and 0.6 g/m^2^ in the RS. For Zn, the addition of each unit resulted in a decrease in native biomass of 2.5 g/m^2^ in IS and an increase of 4.3 g/m^2^ in RS.

## 4 DISCUSSION

The present study provides the first report of earthworm distribution trends along the Rio Doce watershed in Minas Gerais and represents the first widespread replicated survey of earthworm communities (richness, abundance, biomass of native, and invasive species) in riparian forests in Brazil. Across the 30 sampling sites, a total of eight species were recorded, of which two were invasive alien species while the other six were native, with at least two of these representing new species to science. Local species richness (alpha diversity) in the sampling plots was relatively low (0–3 species). Otherwise, these values are comparable to many other sites (*n* = 22) in the Atlantic Forest biome, where most (64%) had only one to two species recorded (Demetrio et al., 2023). In the only previous study with data on earthworm populations in Brazilian riparian forests (J. O. Barreto et al., 2019), 12 earthworm species (eight native, four exotic/invasive) were found in samples taken in five unreplicated sites located in three states (São Paulo, Paraná, Rio Grande do Sul). Similar to this study, several of the native species were considered as new, and remain undescribed so far (J. Barreto, 2019).

Species in the genus *Rhinodrilus* are widely distributed throughout Brazil (Brown & James, 2007). Many of its species are known as “minhocuçus” (eastern indigenous name for large earthworm in Brazil), for their extensive length (>15–20 cm) (Righi, 1999). Some species of this genus may reach > 2 m (Lang et al., 2012). One of the species found in the RS in Rio Casca (*R. motucu*) is a widespread “minhocuçu” species but with a highly scattered distribution from the state of Sergipe (Atlantic Forest in northeastern Brazil) to the state of Mato Grosso (Pantanal wetland) (Righi, 1985). Its large size and deposition of castings on the soil surface (Righi, 1972) indicate that it has important ecosystem functional effects (e.g., porosity, aggregation). Hence, this engineer species absence from IS may imply its sensitivity to the mine tailings, and the potential loss in soil functions in the Rio Casca region. Functional assessments of this kind are essential to adequately inform potential losses in ecosystem services along the Rio Doce watershed. The other *Rhinodrilus* species was sampled in IS in Rio Casca, and Aimorés, and in both IS and RS in Conselheiro Pena, indicating that this species could be more tolerant to this mine tailing deposition.

The 30 known species of *Righiodrilus* occur throughout Latin America (Feijoo & Lavelle, 2023) and are generally small, mostly endogeic. *Righiodrilus* sp. 1 was found in an RS in Conselheiro Pena and in the IS in Mariana, Rio Casca, and Ipatinga, indicating its likely greater tolerance to the mine tailings. The two new species, *Righiodrilus* sp. 2 and sp. 3, were found in Mariana (RS and IS) and in an RS in Ipatinga, respectively. Despite being a common genus in the tropical regions, it is only recently that a large number of new species of this genus have been recorded in South America (Celis et al., 2018; Feijoo & Lavelle, 2023; Santos et al., 2017). The small number and mostly juvenile stage of these species means that further sampling efforts in the region are necessary to determine distribution, potential susceptibility to the mine tailings and obtain specimens for taxonomic description.

Earthworm abundance along the watershed was influenced by both temperature and rainfall, with more earthworms (116–173 ind/m^2^) found in the upper (Mariana) than lower (Aimorés) Rio Doce (9–23 ind/m^2^). Rainfall and temperature are considered the most important factors influencing earthworm populations (Phillips et al., 2019; J. Singh et al., 2019). The site with the highest rainfall and milder temperatures (Mariana) exhibited greater earthworm biomass, regardless of whether it was impacted by tailings or not. Only in Aimorés earthworm abundance was lower than in all the other regions sampled, possibly due to the lower usual rainfall and soil moisture (Cemaden, 2023).

The COIA analysis indicated a significant connection between soil properties and earthworm species. The mining tailing altered the properties of the soil, which had a direct impact on the soil biological community. IS presented higher FiS and lower CoS concentration (G. W. Fernandes et al., 2025), which in turn favored the invasive *P. corethrurus*. As observed in many other sites from Atlantic Forest in Brazil (Demetrio et al., 2023), the invasive alien species *P. corethrurus* predominated, representing a minimum of at least 90% of individuals collected at each site and >93% of all individuals found in IS and RS sites overall (Table 1). This species is native to the Guianas (Taheri et al., 2018) and can easily colonize a wide variety of habitats with a range of different soil conditions, including contaminated soils (Zavala‐Cruz et al., 2013), or with low fertility (Lavelle et al., 1987). Colonization by *P. corethrurus* in the absence of other earthworm species and in soils with low organic matter contents can lead to soil compaction, diminish water and air infiltration (Barros et al., 2004; Hallaire et al., 2000), and reduce the populations of other soil organisms (Demetrio et al., 2023); therefore, altering soil functioning, biodiversity, and ecosystem service delivery (Marichal et al., 2014, 2010). Unfortunately, no historical data are available for the studied sites. However, the predominance of this species, coupled with the overall low abundance or absence of native species in the sampling sites, is concerning and suggests the disturbed condition of these forests (Römbke et al., 2009), including those considered well‐preserved remnants of the Atlantic Forest. The ability of *P. corethrurus* to disperse as far as the southernmost regions of Brazil (Santa Catarina) was first noted over 150 years ago, when Müller (1857) described it as the most common earthworm species in the country.

The other invasive alien species, *A. gracilis*, was found in RS plots in Mariana, as well as RS and IS plots in Rio Casca and Ipatinga, confirming the past disturbance history of these forests. This species native to Asia (Gates, 1972) is a rapid colonizer of new sites due to its frequent movement on the soil surface (J. O. Fernandes et al., 2010). This species could have arrived with soil of transplanted trees or ornamental plants in these regions (Brown et al., 2006).

This study highlighted that all physical and chemical soil attributes influenced the total abundance of earthworms, suggesting a strong association between earthworms and their habitat, where they live, forage, and reproduce (Brown & Doube, 2004). In our study, silt was highlighted as an important influence on the invasive earthworm biomass, corroborating the observations of Huerta et al. (2007). For native earthworms, C, Ca, Mg, and eCEC stood out as essential soil properties to increase their biomass. These results reinforce the observations of co‐inertia that indicate that the species found in this study have a preference for more nutritionally rich environments. In the reference environment, there is greater biodiversity and vegetation cover, which likely promotes the availability of organic matter that earthworms rely on. The incorporation of this organic matter into the soil helps reduce the production of organic acids (Haimi & Huhta, 1990) and increases the availability of macronutrients (Reich et al., 2005).

The study also increases our understanding of the influence of mining tailings on earthworm communities. Mine tailings impacted native earthworm biomass, which had lower biomass in IS compared to RS, indicating negative effects of tailings on their foraging, development, and possibly fitness (Miller et al., 2024). Changes in soil properties (as pH and Zn) between RS and IS can have substantially influenced the native earthworm biomass that are adapted to acid soil and richer in Zn. Moreover, more alkaline and nutritionally poor soils, such as those found in IS, can have a negative effect on native earthworm survival and performance (Edwards & Arancon, 2022; S. Singh et al., 2020; Tamartash & Ehsani, 2021).

The abundance and biomass of the invasive species *P. corethrurus* were not affected by the impact condition, suggesting that high tolerance by earthworm species, as already observed by Ganihar (2003). Despite the plasticity of *P. corethurus*, some soil parameters in the Rio Doce watershed (such as pH, P, Ca, Mg, eCEC, Bsat, C, S, Cu, Mn, Fe, Zn, and silt) had significant effects on its abundance. Most of the correlations between these soil characteristics and the invasive earthworm abundance found in IS were the opposite to those found in the reference condition. These results show that the Samarco's Fundão dam collapse led to changes in the relationship between soil and invasive alien earthworms. Controlled laboratory experiments and further long term studies are suggested to better understand the implications of these impacts and deepen our understanding of the interaction between soil variables and earthworm dynamics.

## AUTHOR CONTRIBUTIONS

**Herlon Nadolny**: Data curation; investigation; methodology; writing—original draft; writing—review and editing. **Yumi Oki**: Conceptualization; data curation; investigation; methodology; project administration; visualization; writing—original draft; writing—review and editing. **Walisson Kenedy‐Siqueira**: Data curation; formal analysis; investigation; visualization; writing—original draft; writing—review and editing. **Marcos P. Santos**: Data curation; investigation; methodology; writing—original draft; writing—review and editing. **Luis M. Hernández‐García**: Data curation; investigation; methodology; writing—original draft; writing—review and editing. **Daniel Negreiros**: Data curation; formal analysis; investigation; writing—original draft; writing—review and editing. **João C. G. Figueiredo**: Investigation; methodology; writing—original draft. **Fernando F. Goulart**: Formal analysis; writing—original draft. **George G. Brown**: Conceptualization; investigation; writing—original draft; writing—review and editing. **Geraldo W. Fernandes**: Conceptualization; funding acquisition; investigation; methodology; supervision; writing—original draft; writing—review and editing.

## CONFLICT OF INTEREST STATEMENT

The authors declare no conflicts of interest.

## Supporting information



Supplementary material.
