# Supplementary material for: Devastating Rio Doce mining disaster sends shockwaves through earthworm populations
Source: J Environ Qual. 2025 Jul 1;54(6):1788–803. doi: 10.1002/jeq2.70056 (PMC12593309; doi:10.1002/jeq2.70056)
Supplement: Supplementary file 1 — Supplementary material. [file JEQ2-54-1788-s001.docx]

**Supplementary Material**

**DEVASTATING RIO DOCE MINING DISASTER SENDS SHOCKWAVES THROUGH EARTHWORM POPULATIONS**

**Herlon Nadolny, Yumi Oki, Walisson Kenedy-Siqueira, Marcos P. Santos, Luis M. Hernández-García, João C. G. Figueiredo, Daniel Negreiros, George G. Brown, Fernando Goulart, Geraldo W. Fernandes**

**
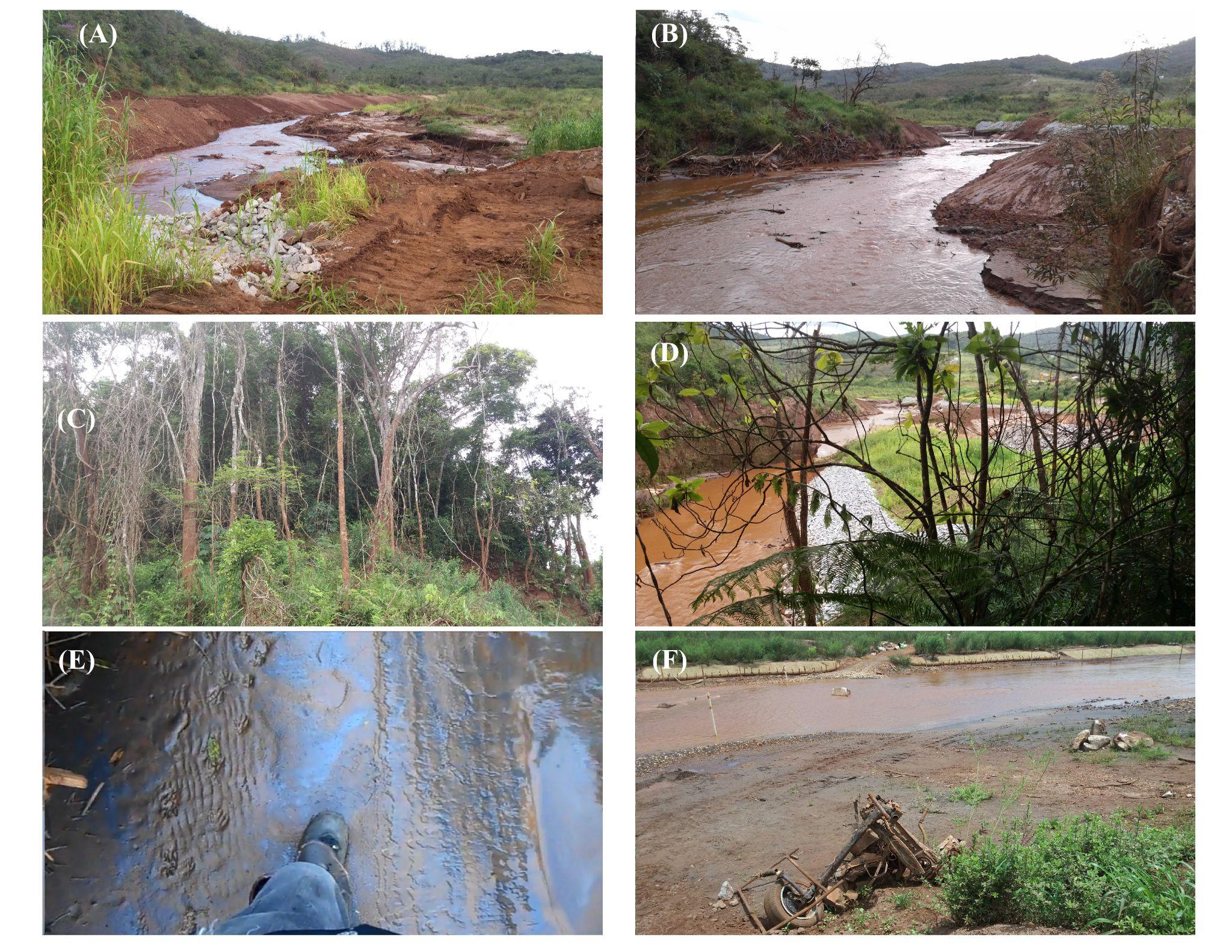
**

**Fig. S1.** Photos of the areas impacted by the rupture of the Fundão Dam in Santa Rita Durão district, Mariana, Minas Gerais, Brazil, taken in 2017: (A)- (D): Effect of the disaster on vegetation. (E) - (F):

Images of the topsoil after the tailings passage. The tailings material released from the Fundão Dam exhibits geomechanical behavior similar to that of quicksand, characterized by high moisture content and low intergranular cohesion between sand and silt particles, which increases its susceptibility to fluidization under stress.


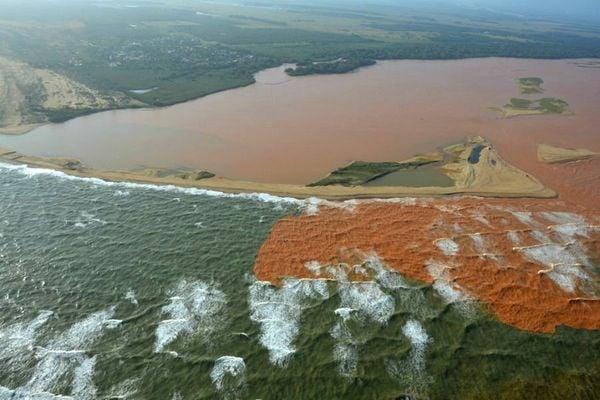


**Fig. S2.** Mud from the Fundão Mariana dam reaches the sea off the coast of Espírito Santo.

**
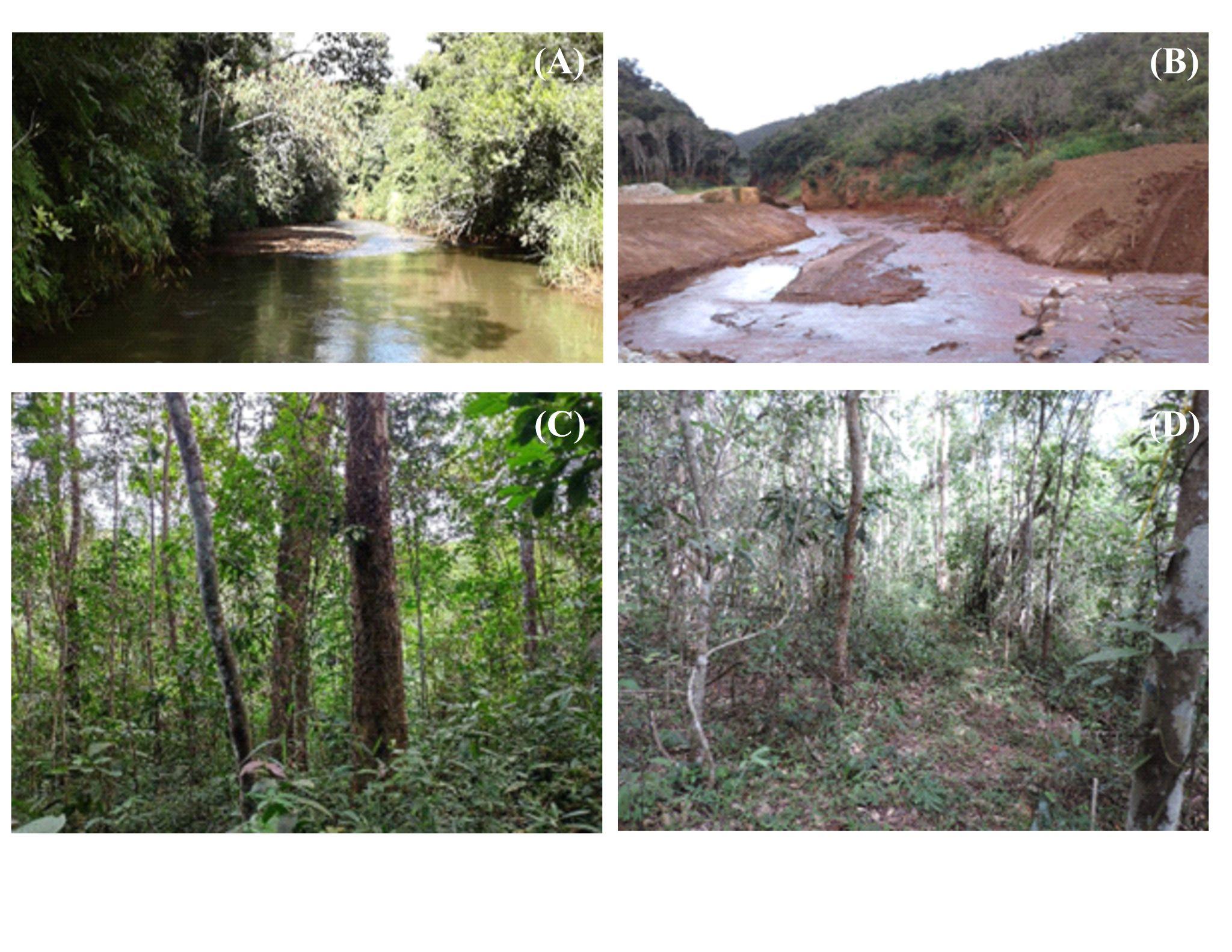
**

**Fig. S3.** Images captured in 2017 in Mariana, two years after the accident: (A) Reference area (section of the Gualaxo do Norte River unaffected by the rupture of the Doce River dam); (B) Impacted area (section of the Gualaxo do Norte River affected by the disaster). Images from the interior of the Riparian Forest, in Mariana, captured in 2023, eight years after the accident: (C) Reference area; (D) Impacted area.


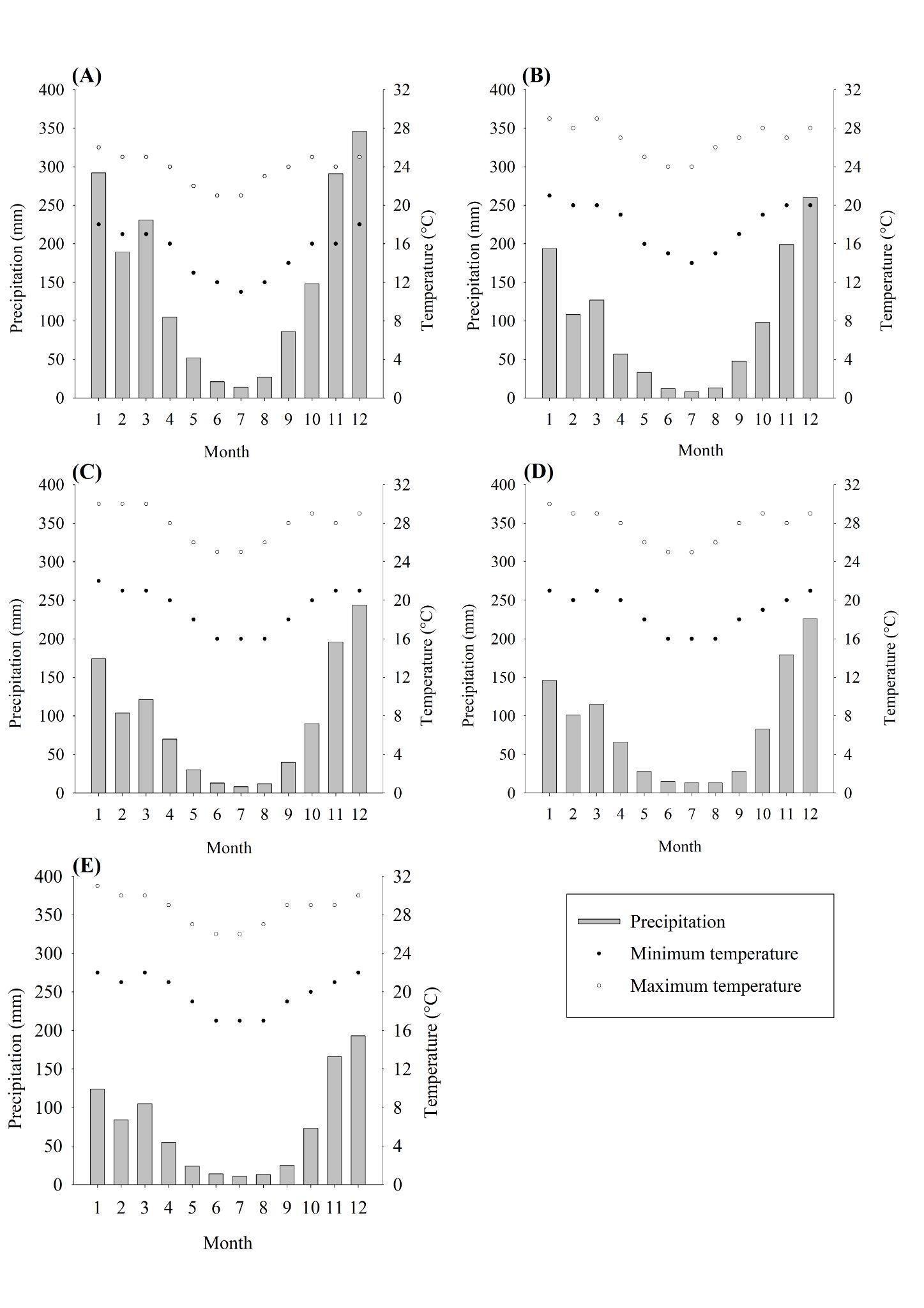


**Fig. S4.** Average of month of precipitation (mm) and minimum and maximum temperature (°C) data for the last 30 years (1993-2023) of the 5 regions along the Rio Doce watershed, Brazil: (A) Mariana; (B) Rio Casca; (C) Ipatinga; (D) Conselheiro Pena; (E) Aimorés. Rainfall and temperature data were obtained from Climatempo (<https://www.climatempo.com.br/climatologia/3703/datas-mg>)


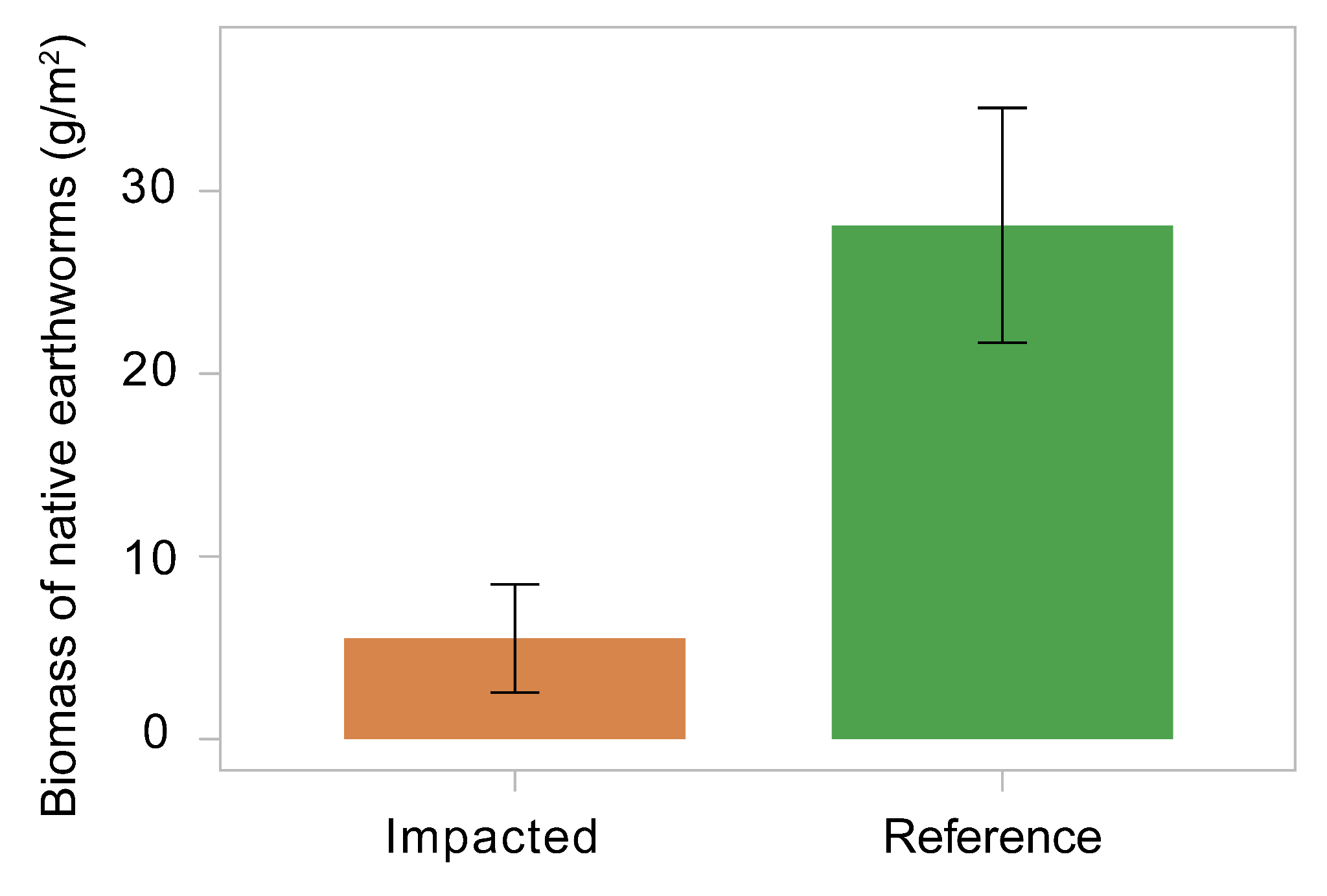


**Fig. S5.** Biomass of native earthworms (average ± standard error) in impacted sites and reference sites (p < 0.05) along Rio Doce watershed.

**Table S1.** Coordinates, Koppen climate classification of sites impacted or not (reference) by mine tailings in riparian forests of five regions (1. Mariana, 2. Rio Casca, 3. Ipatinga, 4. Conselheiro Pena, 5. Aimorés) along the Rio Doce watershed in the state of Minas Gerais, Brazil.

| Region | Municipality | District | Tailings | Latitude | Longitude | Koppen Climate Classification |
| --- | --- | --- | --- | --- | --- | --- |
| 1. Mariana | Mariana | Santa Rita Durão | Impacted | 20°15'25.0"S | 43°25'00.4"W | Cwa |
|  | Mariana | Santa Rita Durão | Impacted | 20°14'25.3"S | 43°19'48.5"W | Cwa |
|  | Mariana | Monsenhor Horta | Impacted | 20°18'03.7"S | 43°13'38.1"W | Cwa |
|  | Mariana | Santa Rita Durão | Reference | 20°16'34.0"S | 43°25'50.0"W | Cwa |
|  | Mariana | Camargos | Reference | 20°17'07.3"S | 43°23'48.5"W | Cwa |
|  | Mariana | Monsenhor Horta | Reference | 20°18'17.0"S | 43°13'11.4"W | Cwa |
| 2. Rio Casca | Rio Casca | Jurumirim | Impacted | 20°04'11.5"S | 42°46'25.5"W | Aw |
|  | Rio Casca | Jurumirim | Impacted | 20°06'03.9"S | 42°45'24.6"W | Aw |
|  | Rio Casca | Jurumirim | Impacted | 20°00'43.7"S | 42°44'34.8"W | Aw |
|  | Santa Cruz do Escalvado | Zito Soares | Reference | 20°08'06.6"S | 42°45'32.8"W | Cwa |
|  | Rio Casca | Jurumirim | Reference | 20°03'11.7"S | 42°45'39.8"W | Aw |
|  | Sem Peixe | São Bartolomeu de Sem Peixe | Reference | 20°00'29.1"S | 42°46'01.3"W | Aw |
| 3. Ipatinga | Bom Jesus do Galho | Revés do Belém | Impacted | 19°34'23.7"S | 42°29'49.5"W | Cwa |
|  | Bom Jesus do Galho | Revés do Belém | Impacted | 19°41'06.0"S | 42°30'04.8"W | Cwa |
|  | Marliéria | - | Impacted | 19°42'29.7"S | 42°29'57.9"W | Aw |
|  | Bom Jesus do Galho | Revés do Belém | Reference | 19°38'53.8"S | 42°28'50.0"W | Cwa |
|  | Bom Jesus do Galho | Revés do Belém | Reference | 19°34'32.2"S | 42°31'19.2"W | Cwa |
|  | Bom Jesus do Galho | Revés do Belém | Reference | 19°33'30.4"S | 42°31'25.6"W | Cwa |
| 4. Conselheiro Pena | Conselheiro Pena | - | Impacted | 19°08'14.2"S | 41°28'19.7"W | Aw |
|  | Conselheiro Pena | - | Impacted | 19°07'08.5"S | 41°29'02.8"W | Aw |
|  | Conselheiro Pena | Barra do Cuieté | Impacted | 19°05'30.1"S | 41°31'01.6"W | Aw |
|  | Conselheiro Pena | Penha do Norte | Reference | 19°06'51.4"S | 41°28'33.3"W | Aw |
|  | Conselheiro Pena | - | Reference | 19°10'10.6"S | 41°26'32.8"W | Aw |
|  | Conselheiro Pena | - | Reference | 19°11'17.6"S | 41°29'35.1"W | Aw |
| 5. Aimorés | Resplendor | - | Impacted | 19°17'33.8"S | 41°16'07.9"W | Aw |
|  | Resplendor | - | Impacted | 19°21'06.4"S | 41°13'18.7"W | Aw |
|  | Itueta | - | Impacted | 19°21'23.6"S | 41°11'28.0"W | Aw |
|  | Itueta | - | Reference | 19°23'37.1"S | 41°08'00.4"W | Aw |
|  | Itueta | - | Reference | 19°23'37.9"S | 41°06'27.3"W | Aw |
|  | Santa Rita do Itueto | - | Reference | 19°29'16.0"S | 41°20'16.3"W | Aw |

*Note*: Aw - Tropical savanna climate, Cwa -Warm temperate with dry winter and hot summer

Table S2. Daily rainfall data for 30 days prior to earthworm collection obtained from CEMADEN weather stations closest to the three reference sites (RS: RS1, RS2, RS3) and three impacted sites (IS: IS1, IS2, IS3) of Mariana, Rio Casca, Ipatinga, Conselheiro Pena, Aimores.

| Name of the weather station | Locality | Site | Precipitation (mm) | |
| --- | --- | --- | --- | --- |
|  |  |  | Day | 30 days before the collection period in each region |
| Vila Maquiné - Mariana - MG | Mariana | RS1, RS2, RS3, IS1, IS2, IS3 | 1 | 0.02 |
|  |  |  | 2 | 0 |
|  |  |  | 3 | 0 |
|  |  |  | 4 | 0 |
|  |  |  | 5 | 0 |
|  |  |  | 6 | 0 |
|  |  |  | 7 | 0.42 |
|  |  |  | 8 | 0.14 |
|  |  |  | 9 | 0.25 |
|  |  |  | 10 | 0.05 |
|  |  |  | 11 | 0 |
|  |  |  | 12 | 0.01 |
|  |  |  | 13 | 0.14 |
|  |  |  | 14 | 0.88 |
|  |  |  | 15 | 0.01 |
|  |  |  | 16 | 0 |
|  |  |  | 17 | 0 |
|  |  |  | 18 | 0 |
|  |  |  | 19 | 0.04 |
|  |  |  | 20 | 0 |
|  |  |  | 21 | 0 |
|  |  |  | 22 | 0 |
|  |  |  | 23 | 0 |
|  |  |  | 24 | 0 |
|  |  |  | 25 | 0 |
|  |  |  | 26 | 0 |
|  |  |  | 27 | 0 |
|  |  |  | 28 | 0.9 |
|  |  |  | 29 | 0.11 |
|  |  |  | 30 | 0 |
|  |  |  | 31 |  |
| Centro - Rio Casca - MG | Rio Casca | RS1, RS2, RS3, IS1, IS2, IS3 | 1 | 0 |
|  |  |  | 2 | 0.06 |
|  |  |  | 3 | 0.07 |
|  |  |  | 4 | 0 |
|  |  |  | 5 | 0 |
|  |  |  | 6 | 0 |
|  |  |  | 7 | 0 |
|  |  |  | 8 | 0.26 |
|  |  |  | 9 | 0.06 |
|  |  |  | 10 | 0 |
|  |  |  | 11 | 0 |
|  |  |  | 12 | 0 |
|  |  |  | 13 | 0 |
|  |  |  | 14 | 0.41 |
|  |  |  | 15 | 0.02 |
|  |  |  | 16 | 0 |
|  |  |  | 17 | 0 |
|  |  |  | 18 | 0 |
|  |  |  | 19 | 0.07 |
|  |  |  | 20 | 0.01 |
|  |  |  | 21 | 0 |
|  |  |  | 22 | 0 |
|  |  |  | 23 | 0 |
|  |  |  | 24 | 0 |
|  |  |  | 25 | 0 |
|  |  |  | 26 | 0 |
|  |  |  | 27 | 0 |
|  |  |  | 28 | 0.10 |
|  |  |  | 29 | 0.02 |
|  |  |  | 30 | 0 |
|  |  |  | 31 |  |
| Centro Norte - Timóteo - MG | Ipatinga | RS1, RS2, RS3, IS1, IS2, IS3 | 1 | 0 |
|  |  |  | 2 | 0.10 |
|  |  |  | 3 | 0.06 |
|  |  |  | 4 | 0 |
|  |  |  | 5 | 0 |
|  |  |  | 6 | 0 |
|  |  |  | 7 | 0 |
|  |  |  | 8 | 0.28 |
|  |  |  | 9 | 0.05 |
|  |  |  | 10 | 0.06 |
|  |  |  | 11 | 0 |
|  |  |  | 12 | 0 |
|  |  |  | 13 | 0 |
|  |  |  | 14 | 0.14 |
|  |  |  | 15 | 0.05 |
|  |  |  | 16 | 0 |
|  |  |  | 17 | 0 |
|  |  |  | 18 | 0 |
|  |  |  | 19 | 0.19 |
|  |  |  | 20 | 0.06 |
|  |  |  | 21 | 0.01 |
|  |  |  | 22 | 0 |
|  |  |  | 23 | 0 |
|  |  |  | 24 | 0 |
|  |  |  | 25 | 0 |
|  |  |  | 26 | 0 |
|  |  |  | 27 | 0.04 |
|  |  |  | 28 | 0.07 |
|  |  |  | 29 | 0.33 |
|  |  |  | 30 | 0 |
|  |  |  | 31 |  |
| Centro - Conselheiro Pena - MG | Conselheiro Pena | RS1, RS2, RS3, IS1, IS2, IS3 | 1 | 0 |
|  |  |  | 2 | 0.07 |
|  |  |  | 3 | 0 |
|  |  |  | 4 | 0 |
|  |  |  | 5 | 0.05 |
|  |  |  | 6 | 0.01 |
|  |  |  | 7 | 0 |
|  |  |  | 8 | 0 |
|  |  |  | 9 | 0.02 |
|  |  |  | 10 | 0 |
|  |  |  | 11 | 0 |
|  |  |  | 12 | 0 |
|  |  |  | 13 | 0 |
|  |  |  | 14 | 0 |
|  |  |  | 15 | 0.28 |
|  |  |  | 16 | 0.50 |
|  |  |  | 17 | 0 |
|  |  |  | 18 | 0 |
|  |  |  | 19 | 0.27 |
|  |  |  | 20 | 0.01 |
|  |  |  | 21 | 0 |
|  |  |  | 22 | 0 |
|  |  |  | 23 | 0 |
|  |  |  | 24 | 0 |
|  |  |  | 25 | 0 |
|  |  |  | 26 | 0 |
|  |  |  | 27 | 0 |
|  |  |  | 28 | 0 |
|  |  |  | 29 | 0.50 |
|  |  |  | 30 | 0.02 |
|  |  |  | 31 |  |
| Igrejinha - Aimorés - MG | Aimorés | RS1, RS2, RS3 | 1 | 0 |
|  |  |  | 2 | 0 |
|  |  |  | 3 | 0 |
|  |  |  | 4 | 0 |
|  |  |  | 5 | 0 |
|  |  |  | 6 | 0 |
|  |  |  | 7 | 0.11 |
|  |  |  | 8 | 0.01 |
|  |  |  | 9 | 0 |
|  |  |  | 10 | 0 |
|  |  |  | 11 | 0 |
|  |  |  | 12 | 0 |
|  |  |  | 13 | 0 |
|  |  |  | 14 | 0 |
|  |  |  | 15 | 0.02 |
|  |  |  | 16 | 0.01 |
|  |  |  | 17 | 0 |
|  |  |  | 18 | 0.01 |
|  |  |  | 19 | 0 |
|  |  |  | 20 | 0 |
|  |  |  | 21 | 0 |
|  |  |  | 22 | 0 |
|  |  |  | 23 | 0 |
|  |  |  | 24 | 0 |
|  |  |  | 25 | 0 |
|  |  |  | 26 | 0 |
|  |  |  | 27 | 0 |
|  |  |  | 28 | 0 |
|  |  |  | 29 | 0 |
|  |  |  | 30 | 0 |
|  |  |  | 31 | 0 |
| Centro - Resplendor - - MG | Aimorés | IS1, IS2, IS3 | 1 | 0 |
|  |  |  | 2 | 0 |
|  |  |  | 3 | 0 |
|  |  |  | 4 | 0 |
|  |  |  | 5 | 0 |
|  |  |  | 6 | 0 |
|  |  |  | 7 | 0.81 |
|  |  |  | 8 | 0.03 |
|  |  |  | 9 | 0 |
|  |  |  | 10 | 0 |
|  |  |  | 11 | 0 |
|  |  |  | 12 | 0 |
|  |  |  | 13 | 0 |
|  |  |  | 14 | 0 |
|  |  |  | 15 | 0.03 |
|  |  |  | 16 | 0.11 |
|  |  |  | 17 | 0.01 |
|  |  |  | 18 | 0.01 |
|  |  |  | 19 | 0 |
|  |  |  | 20 | 0 |
|  |  |  | 21 | 0 |
|  |  |  | 22 | 0 |
|  |  |  | 23 | 0 |
|  |  |  | 24 | 0 |
|  |  |  | 25 | 0 |
|  |  |  | 26 | 0 |
|  |  |  | 27 | 0 |
|  |  |  | 28 | 0 |
|  |  |  | 29 | 0 |
|  |  |  | 30 | 0 |
|  |  |  | 31 | 0 |

*Note*: Designations: RS-reference sites, IS -impacted sites, RS1, RS2, RS3 - reference site 1, reference site 2, reference site 3, IS1, IS2, IS3 -impacted sites 1, impacted site 2, impacted site 3

Table S3. Chemical and physical soil properties of the sites sampled along the Rio Doce watershed. The first letter of the site represents the initials of the region's first name: M= Mariana, R= Rio Casca, I= Ipatinga, C= Conselheiro Pena, A= Aimores. The second letter of the site represents the presence or absence of mine tailings, where I= Impacted sites; R= Reference sites. The number after the second letter of the site represents the site number: 1, 2, 3. For each condition, 3 sites were established.

| Site | **pH** | **P** | **K** | **Ca** | **Mg** | **Al** | **Bsat** | **C** | **S** | **Cu** | **Mn** | **Fe** | **Zn** | **Cs** | **Fs** | **Silt** | **Clay** | **DP** |
| --- | --- | --- | --- | --- | --- | --- | --- | --- | --- | --- | --- | --- | --- | --- | --- | --- | --- | --- |
| MR1 | 5.23 ± 0.12 | 2.4 ± 0.2 | 0.13 ± 0.01 | 1.99 ± 0.24 | 0.87 ± 0.07 | 0.12 ± 0.04 | 42.8 ± 4.31 | 2.05 ± 0.20 | 3.74 ± 1.42 | 1.27 ± 0.13 | 264 ± 30.5 | 50.2 ± 9.89 | 2.57 ± 0.23 | 0.100 ± 0.02 | 0.38 ± 0.03 | 0.34 ± 0.02 | 0.18 ± 0.01 | 2.89 ± 0.04 |
| MR2 | 4.60 ± 0.07 | 2.4 ± 0.2 | 0.08 ± 0.01 | 0.89 ± 0.07 | 0.38 ± 0.03 | 0.59 ± 0.06 | 24.13 ± 2.88 | 1.38 ± 0.07 | 8.48 ± 1.25 | 0.58 ± 0.07 | 141 ± 19 | 87,5 ± 12.8 | 1,02 ± 0.26 | 0.14 ± 0.02 | 0.41 ± 0.03 | 0.26 ± 0.03 | 0.18 ± 0.02 | 2.89 ± 0.03 |
| MR3 | 3.88 ± 0.05 | 4.2 ± 0.4 | 0.14 ± 0.01 | 0.33 ± 0.19 | 0.28 ± 0.07 | 1.85 ± 0.09 | 5.74 ± 1.7 | 4.09 ± 0.21 | 18.2 ± 2.75 | 1.29 ± 0.21 | 25.9 ± 4.72 | 112 ± 11.0 | 1.41 ± 0.17 | 0.20 ± 0.02 | 0.17 ± 0.02 | 0.17 ± 0.02 | 0.46 ± 0.01 | 2.42 ± 0.03 |
| MI1 | 5.96 ± 0.10 | 2.6 ± 0.3 | 0.08 ± 0.02 | 0.94 ± 0.06 | 0.23 ± 0.03 | 0.0000 | 54.7 ± 2.93 | 0.36 ± 0.04 | 7.60 ± 0.55 | 1.30 ± 0.12 | 180.02 ± 18.4 | 137.39 ± 15.1 | 0.99 ± 0.07 | 0.1 ± 0.01 | 0.51 ± 0.03 | 0.29 ± 0.03 | 0.10 ± 0.01 | 2.96 ± 0.03 |
| MI2 | 5.37 ± 0.14 | 3.5 ± 0.7 | 0.12 ± 0.02 | 0.91 ± 0.12 | 0.21 ± 0.03 | 0.13 ± 0.07 | 42.6 ± 4.45 | 0.60 ± 0.13 | 10.13 ± 2.27 | 0.70 ± 0.07 | 104 ± 10.7 | 95.08 ± 28.6 | 0.80 ± 0.05 | 0.16 ± 0.03 | 0.38 ± 0.04 | 0.30 ± 0.03 | 0.16 ± 0.03 | 2.92 ± 0.04 |
| MI3 | 5.73 ± 0.10 | 4.2 ± 0.4 | 0.15 ± 0.02 | 1.03 ± 0.09 | 0.32 ± 0.04 | 0.00 | 56.3 ± 2.68 | 0.30 ± 0.03 | 4.14 ± 0.63 | 0.94 ± 0.04 | 121 ± 7.26 | 87 ± 11.4 | 1.20 ± 0.25 | 0.08 ± 0.01 | 0.44 ± 0.02 | 0.37 ± 0.02 | 0.11 ± 0.01 | 3.00 ± 0.02 |
| RR1 | 5.2 ± 0.15 | 3.7 ± 0.7 | 0.08 ± 0.01 | 1.58 ± 0.14 | 0.6 ± 0.05 | 0.11 ± 0.04 | 49.9 ± 4.35 | 0.87 ± 0.16 | 7.99 ± 1.67 | 1.68 ± 0.08 | 60.8 ± 4.46 | 70.8± 14.4 | 2.16 ± 0.14 | 0.47 ± 0.04 | 0.29 ± 0.06 | 0.07 ± 0.01 | 0.17 ± 0.02 | 2.60 ± 0.02 |
| RR2 | 4.16 ± 0.09 | 3.7 ± 0.4 | 0.20 ± 0.02 | 1.32 ± 0.25 | 0.68 ± 0.10 | 0.87 ± 0.12 | 23.5 ± 3.13 | 2.14 ± 0.14 | 19.9 ± 1.49 | 2.00 ± 0.09 | 78.9 ± 13.3 | 71.2 ± 7.48 | 1.86 ± 0.18 | 0.46 ± 0.02 | 0.15 ± 0.01 | 0.07 ± 0.01 | 0.33 ± 0.01 | 2.51 ± 0.02 |
| RR3 | 5.42 ± 0.11 | 2.6 ± 0.5 | 0.22 ± 0.02 | 4.03 ± 0.62 | 2.32 ± 0.23 | 0.03 ± 0.02 | 63.2 ± 3.00 | 1.83 ± 0.11 | 5.05 ± 1.48 | 2.15 ± 0.18 | 77.9 ± 6.25 | 83.9 ± 17.4 | 3.54 ± 0.66 | 0.19 ± 0.02 | 0.16 ± 0.01 | 0.24 ± 0.01 | 0.41 ± 0.02 | 2.37 ± 0.02 |
| RI1 | 5.85 ± 0.09 | 12 ± 1.0 | 0.12 ± 0.02 | 2.38 ± 0.22 | 0.91 ± 0.12 | 0.01 ± 0.01 | 69.67 ± 1.98 | 0.66 ± 0.12 | 2.27 ± 0.52 | 1.75 ± 0.23 | 98.3 ± 7.8 | 151 ± 16.4 | 2.44 ± 0.17 | 0.30 ± 0.05 | 0.49 ± 0.03 | 0.10 ± 0.02 | 0.11 ± 0.01 | 2.70 ± 0.02 |
| RI2 | 5.65 ± 0.09 | 9.1 ± 0.3 | 0.16 ± 0.01 | 2.79 ± 0.25 | 0.90 ± 0.08 | 0.00 | 70.2 ± 1.89 | 0.89 ± 0.12 | 3.02 ± 0.62 | 2.43 ± 0.20 | 124 ± 11.3 | 190 ± 15.6 | 2.89 ± 0.30 | 0.13 ± 0.02 | 0.55 ± 0.03 | 0.18 ± 0.01 | 0.14 ± 0.02 | 2.65 ± 0.02 |
| RI3 | 5.71 ± 0.12 | 7.5 ± 0.3 | 0.09 ± 0.01 | 2.35 ± 0.19 | 0.82 ± 0.07 | 0.02 ± 0.01 | 62. ± 2.11 | 0.83 ± 0.08 | 3.3 ± 0.59 | 2.28 ± 0.23 | 107 ± 6.18 | 196 ± 19.6 | 2.56 ± 0.17 | 0.19 ± 0.04 | 0.53 ± 0.03 | 0.15 ± 0.02 | 0.13 ± 0.02 | 2.66 ± 0.02 |
| IR1 | 5.73 ± 0.07 | 6.5± 0.3 | 0.17 ± 0.01 | 2.80 ± 0.12 | 1.33 ± 0.04 | 0.00 | 72.2 ± 1.86 | 0.79 ± 0.05 | 15.9 ± 7.16 | 2.58 ± 0.12 | 156 ± 9.09 | 216 ± 17.2 | 3.971 ± 0.16 | 0.14 ± 0.02 | 0.54 ± 0.01 | 0.15 ± 0.01 | 0.17 ± 0.01 | 2.64 ± 0.02 |
| IR2 | 5.39 ± 0.10 | 3.8 ± 0.3 | 0.13 ± 0.01 | 2.28 ± 0.21 | 1.55 ± 0.16 | 0.04 ± 0.02 | 57.88 ± 1.75 | 1.24 ± 0.18 | 4.91 ± 0.72 | 3.50 ± 0.29 | 98.8 ± 10.3 | 234.45 ± 30.2 | 3.94 ± 0.33 | 0.03 ± 0.01 | 0.43 ± 0.06 | 0.25 ± 0.03 | 0.29 ± 0.04 | 2.50 ± 0.04 |
| IR3 | 5.40 ± 0.06 | 1.9± 0.2 | 0.18 ± 0.01 | 2.39 ± 0.16 | 1.94 ± 0.09 | 0.01 ± 0.01 | 58.5 ± 1.27 | 1.73 ± 0.19 | 8.43 ± 0.64 | 1.75 ± 0.10 | 80.8 ± 5.96 | 55.8 ± 9.4 | 3.42 ± 0.21 | 0.20 ± 0.01 | 0.27 ± 0.03 | 0.21 ± 0.01 | 0.33 ± 0.02 | 2.47 ± 0.04 |
| II1 | 5.21 ± 0.07 | 3.17 ± 0.3 | 0.22 ± 0.02 | 2.88 ± 0.17 | 1.54 ± 0.08 | 0.03 ± 0.01 | 62.31 ± 1.42 | 1.59 ± 0.13 | 7.16 ± 0.84 | 2.45 ± 0.16 | 126 ± 10.9 | 152 ± 14.3 | 3.50 ± 0.23 | 0.10 ± 0.03 | 0.39 ± 0.05 | 0.21 ± 0.02 | 0.29 ± 0.02 | 2.50 ± 0.02 |
| II2 | 5.23 ± 0.07 | 5.5 ± 0.4 | 0.17 ± 0.02 | 1.90 ± 0.16 | 0.93 ± 0.09 | 0.010 ± 0.01 | 63.0 ± 1.44 | 0.69 ± 0.10 | 5.01 ± 0.23 | 1.93 ± 0.16 | 99.5± 10.8 | 180 ± 8.71 | 2.52 ± 0.18 | 0.26 ± 0.03 | 0.56 ± 0.03 | 0.08 ± 0.01 | 0.11 ± 0.01 | 2.67 ± 0.02 |
| II3 | 4.85 ± 0.13 | 4.4 ± 0.4 | 0.16 ± 0.01 | 1.53 ± 0.16 | 0.80 ± 0.07 | 0.20 ± 0.07 | 49.24 ± 3.31 | 0.87 ± 0.10 | 8.84 ± 0.9 | 1.84 ± 0.14 | 89 ± 5.91 | 181 ± 19.7 | 2.88 ± 0.34 | 0.21 ± 0.04 | 0.54 ± 0.05 | 0.10 ± 0.01 | 0.15 ± 0.01 | 2.64 ± 0.02 |
| CR1 | 5.33 ± 0.04 | 1.2 ± 0.2 | 0.29 ± 0.02 | 1.45 ± 0.17 | 1.12 ± 0.08 | 0.04 ± 0.01 | 55.49 ± 2.0 | 0.88 ± 0.08 | 8.85 ± 0.47 | 0.74 ± 0.04 | 37.5 ± 3.49 | 29.9 ± 1.87 | 1.20 ± 0.10 | 0.44 ± 0.01 | 0.25 ± 0.01 | 0.09 ± 0.00 | 0.22 ± 0.01 | 2.68 ± 0.01 |
| CR2 | 5.67 ± 0.04 | 0.61 ± 0.1 | 0.24 ± 0.02 | 3.21 ± 0.19 | 1.11 ± 0.09 | 0.01 ± 0.01 | 61.9 ± 1.13 | 1.63 ± 0.12 | 5.67 ± 0.41 | 0.23 ± 0.01 | 38.4 ± 2.2 | 19.9 ± 1.71 | 1.6 ± 0.21 | 0.45 ± 0.02 | 0.11 ± 0.01 | 0.11 ± 0.01 | 0.32 ± 0.03 | 2.57 ± 0.03 |
| CR3 | 6.12 ± 0.10 | 1.35 ± 0.3 | 0.26 ± 0.02 | 8.24 ± 0.40 | 1.46 ± 0.11 | 0.004 ± 0.004 | 79.7± 1.12 | 1.69 ± 0.13 | 1.95 ± 0.40 | 0.36 ± 0.02 | 51.67 ± 2.69 | 47.42 ± 9.98 | 1.83 ± 0.16 | 0.38 ± 0.03 | 0.20 ± 0.06 | 0.10 ± 0.01 | 0.33 ± 0.03 | 2.55 ± 0.02 |
| CI1 | 5.55 ± 0.19 | 4.6 ± 0.6 | 0.25 ± 0.03 | 3.72 ± 0.40 | 1.84 ± 0.23 | 0.122 ± 0.04 | 65.05 ± 3.57 | 1.71 ± 0.14 | 6.01 ± 0.72 | 2.68 ± 0.27 | 125 ± 32.5 | 321 ± 74.6 | 3.97 ± 0.54 | 0.0412 ± 0.03 | 0.14 ± 0.02 | 0.33 ± 0.04 | 0.48 ± 0.03 | 2.53 ± 0.04 |
| CI2 | 5.60 ± 0.08 | 7.7 ± 1.0 | 0.40 ± 0.03 | 5.68 ± 0.54 | 2.12 ± 0.18 | 0.014 ± 0.01 | 70.83 ± 2.33 | 2.51 ± 0.34 | 3.27 ± 0.80 | 0.98 ± 0.14 | 75.02 ± 8.32 | 60.25 ± 7.65 | 4.63 ± 0.70 | 0.253 ± 0.05 | 0.174 ± 0.02 | 0.12 ± 0.02 | 0.46 ± 0.04 | 2.47 ± 0.02 |
| CI3 | 6.16 ± 0.37 | 10 ± 4.7 | 0.49 ± 0.12 | 4.59 ± 0.66 | 1.55 ± 0.12 | 0.04 ± 0.01 | 69.7 ± 3.96 | 1.90 ± 0.17 | 51.8 ± 42.3 | 1.79 ± 0.12 | 59.9 ± 4.00 | 84.5 ± 6.41 | 3.40 ± 0.34 | 0.03 ± 0.01 | 0.09 ± 0.02 | 0.27 ± 0.01 | 0.61 ± 0.02 | 2.58 ± 0.01 |
| AR1 | 5.90 ± 0.10 | 5.6 ± 0.4 | 0.37 ± 0.02 | 4.30 ± 0.42 | 2.15 ± 0.24 | 0 ± 0 | 72.5 ± 3.07 | 2.17 ± 0.16 | 3.43 ± 1.15 | 0.33 ± 0.04 | 48.1 ± 3.67 | 9.67 ± 1.01 | 4.93 ± 0.44 | 0.43 ± 0.02 | 0.19 ± 0.01 | 0.09 ± 0.01 | 0.29 ± 0.02 | 2.61 ± 0.02 |
| AR2 | 6.08 ± 0.06 | 7.5 ± 0.7 | 0.41 ± 0.03 | 4.36 ± 0.38 | 185 ± 0.19 | 0.00 | 76.5 ± 1.6 | 1.81 ± 0.16 | 0.73 ± 0.42 | 0.43 ± 0.04 | 82.0 ± 11.9 | 6.27 ± 0.81 | 4.70 ± 0.38 | 0.48 ± 0.02 | 0.18 ± 0.004 | 0.11 ± 0.004 | 0.24 ± 0.02 | 2.64 ± 0.02 |
| AR3 | 4.45 ± 0.04 | 2.0 ± 0.2 | 0.23 ± 0.01 | 1.37 ± 0.19 | 0.62 ± 0.05 | 0.60 ± 0.07 | 28.43 ± 2.12 | 1.93 ± 0.13 | 18.24 ± 1.1 | 0.29 ± 0.10 | 17.40 ± 3.93 | 80.25 ± 11.4 | 1.12 ± 0.20 | 0.40 ± 0.03 | 0.08 ± 0.01 | 0.10 ± 0.02 | 0.41 ± 0.02 | 2.54 ± 0.03 |
| AI1 | 4.57 ± 0.28 | 0.2± 0.02 | 2.37 ± 0.18 | 0.84 ± 0.10 | 0.02 ± 0.01 | 3.39 ± 0.28 | 0.80 ± 0.12 | 41.9 ± 1.44 | 1.71 ± 0.15 | 55.2 ± 5.2 | 252 ± 21.6 | 2.89 ± 0.21 | 0.15 ± 0.04 | 0.53 ± 0.05 | 0.14 ± 0.03 | 0.17 ± 0.02 | 0.25 ± 0.03 | 2.45 ± 0.04 |
| AI2 | 3.15 ± 0.5 | 0.3 ± 0.03 | 7.32 ± 1.1 | 2.63 ± 0.35 | 0.09 ± 0.04 | 10.3 ± 1.34 | 2.47 ± 0.21 | 38.3 ± 0.91 | 1.17 ± 0.14 | 61.7 ± 9.11 | 45.9 ± 6.54 | 2.79 ± 0.35 | 0.17 ± 0.03 | 0.28 ± 0.03 | 0.14 ± 0.02 | 0.41 ± 0.04 | 0.24 ± 0.04 | 2.38 ± 0.04 |
| AI3 | 16.3 ± 5.84 | 0.3 ± 0.02 | 3.38 ± 0.28 | 1.76 ± 0.19 | 0.008 ± 0.01 | 5.44 ± 0.39 | 1.25 ± 0.16 | 45.75 ± 1.29 | 0.75 ± 0.12 | 51.7 ± 4.92 | 54.8 ± 20.1 | 2.30 ± 0.29 | 0.47 ± 0.03 | 0.2 ± 0.03 | 0.06 ± 0.01 | 0.26 ± 0.03 | 0.25 ± 0.03 | 2.50 ± 0.03 |

*Note*: Designations: M -Mariana, R -Rio Casca, I -Ipatinga, C -Conselheiro Pena, A -Aimores, I -Impacted sites, R -Reference sites, MR1-MR3 -Mariana's reference site 1-3, MI1-MI3 -Mariana’s impacted sites 1-3, RR1-RR3 -Rio Casca’s Reference sites 1-3, RI1-RI3 -Rio Casca’s impacted sites 1-3, IR1-IR3 -Ipatinga’s Reference sites 1-3, II1-II3 -Ipatinga’s impacted sites 1-3, CR1-CR3 -Conselheiro Pena’s Reference sites 1-3, CI1-CI3 -Conselheiro Pena’s impacted sites 1-3, AR1-AR3 -Aimores’s Reference sites 1-3, AI1-AI3 -Aimores’s impacted sites 1-3. Soil properties: P -phosphorus (mg/dm^3^), K -potassium (cmolc/dm^3^), Ca -calcium (cmolc/dm^3^), Mg -magnesium ( (cmolc/dm^3^), Al -aluminum ( (cmolc/dm^3^), C -carbon (g/kg), S -sulfur (mg/dm^3^), Cu -copper ((mg/dm^3^), Mn -manganese (mg/dm^3^), Fe -iron ((mg/dm^3^), Zn -zinc ((mg/dm^3^), Bsat -base saturation (%), Cs -coarse sand (kg/kg), Fs -fine sand (kg/kg), Silt (kg/kg), Clay (Kg/kg), PD - Particle density (g/cm^3^).

Table S4. Total and mean abundance (number of individuals/m^2^) per earthworm species and richness (Ric.) of earthworms collected in five riparian forest regions: Mariana (M), Rio Casca (R), Ipatinga (I), Conselheiro Pena (C) and Aimorés (A). In each region, 15 non-impacted sites (reference, R, n=15) and 15 impacted sites (I, n=15) were selected per region. The numbers (1, 2, 3) refer to the three replicate sites in each region.

| Site | Mean abundance per species (number of individuals/m^2^) | | | | | | | | Ric. |
| --- | --- | --- | --- | --- | --- | --- | --- | --- | --- |
|  | Invasive earthworms | | Native earthworms | | | | | |  |
|  | *Pontoscolex* | *Amynthas* | *Rhinodrilus* | | *Righiodrilus* | | | Ocnerodrilidae |  |
|  | *P. corenthurus* | *A. gracilis* | *Rhinodrilus* sp.1 | *R. motucu* | *Righiodrilus* sp.1 | *Righiodrilus* sp.2 | *Righiodrilus* sp.3 | Ocnerodrilidae sp. |  |
| MR1 | 106.67 | 3.33 | 0.00 | 0.00 | 0.00 | 0.00 | 0.00 | 0.00 | 2 |
| MR2 | 81.67 | 0.00 | 0.00 | 0.00 | 0.00 | 3.33 | 0.00 | 0.00 | 2 |
| MR3 | 78.33 | 0.00 | 0.00 | 0.00 | 0.00 | 0.00 | 0.00 | 0.00 | 0 |
| MI1 | 21.67 | 0.00 | 0.00 | 0.00 | 0.00 | 0.00 | 0.00 | 0.00 | 1 |
| MI2 | 98.33 | 1.67 | 0.00 | 0.00 | 1.67 | 1.67 | 0.00 | 0.00 | 4 |
| MI3 | 53.33 | 0.00 | 0.00 | 0.00 | 0.00 | 3.33 | 0.00 | 0.00 | 2 |
| RR1 | 1.67 | 0.00 | 0.00 | 0.00 | 0.00 | 0.00 | 0.00 | 0.00 | 1 |
| RR2 | 50.00 | 0.00 | 0.00 | 0.00 | 0.00 | 0.00 | 0.00 | 0.00 | 0 |
| RR3 | 55.00 | 0.00 | 0.00 | 8.33 | 0.00 | 0.00 | 0.00 | 0.00 | 2 |
| RI1 | 10.00 | 0.00 | 0.00 | 0.00 | 1.67 | 0.00 | 0.00 | 0.00 | 2 |
| RI2 | 18.33 | 1.67 | 0.00 | 0.00 | 0.00 | 0.00 | 0.00 | 0.00 | 2 |
| RI3 | 18.33 | 0.00 | 0.00 | 0.00 | 1.67 | 0.00 | 0.00 | 0.00 | 2 |
| IR1 | 11.67 | 0.00 | 0.00 | 0.00 | 0.00 | 0.00 | 0.00 | 0.00 | 1 |
| IR2 | 15.00 | 0.00 | 0.00 | 0.00 | 0.00 | 0.00 | 0.00 | 0.00 | 1 |
| IR3 | 53.33 | 1.67 | 0.00 | 0.00 | 0.00 | 0.00 | 1.67 | 0.00 | 3 |
| II1 | 63.33 | 0.00 | 0.00 | 0.00 | 0.00 | 0.00 | 0.00 | 3.33 | 2 |
| II2 | 23.33 | 3.33 | 0.00 | 0.00 | 0.00 | 0.00 | 0.00 | 0.00 | 2 |
| II3 | 38.33 | 0.00 | 0.00 | 0.00 | 0.00 | 0.00 | 0.00 | 0.00 | 1 |
| CR1 | 1.67 | 0.00 | 0.00 | 0.00 | 0.00 | 0.00 | 0.00 | 0.00 | 1 |
| CR2 | 0.00 | 0.00 | 5.00 | 0.00 | 1.67 | 0.00 | 0.00 | 0.00 | 2 |
| CR3 | 3.33 | 0.00 | 10.00 | 0.00 | 0.00 | 0.00 | 0.00 | 0.00 | 2 |
| CI1 | 113.33 | 1.67 | 1.67 | 0.00 | 0.00 | 0.00 | 0.00 | 0.00 | 2 |
| CI2 | 0.00 | 0.00 | 5.00 | 0.00 | 0.00 | 0.00 | 0.00 | 0.00 | 1 |
| CI3 | 0.00 | 0.00 | 0.00 | 0.00 | 0.00 | 0.00 | 0.00 | 0.00 | 0 |
| AR1 | 0.00 | 3.33 | 1.67 | 0.00 | 0.00 | 0.00 | 0.00 | 0.00 | 1 |
| AR2 | 0.00 | 0.00 | 0.00 | 0.00 | 0.00 | 0.00 | 0.00 | 0.00 | 0 |
| AR3 | 11.67 | 0.00 | 0.00 | 0.00 | 0.00 | 0.00 | 0.00 | 0.00 | 1 |
| AI1 | 30.00 | 0.00 | 0.00 | 0.00 | 0.00 | 0.00 | 0.00 | 0.00 | 1 |
| AI2 | 0.00 | 0.00 | 0.00 | 0.00 | 0.00 | 0.00 | 0.00 | 0.00 | 0 |
| AI3 | 6.67 | 0.00 | 0.00 | 0.00 | 0.00 | 0.00 | 0.00 | 0.00 | 1 |
| **Total** | 966.00 | 18.67 | 26.33 | 12.33 | 11.67 | 14.33 | 8.67 | 11.33 | 8 |

*Note*: Designations: M -Mariana, R -Rio Casca, I -Ipatinga, C -Conselheiro Pena, A -Aimores, I -Impacted sites, R -Reference sites, MR1-MR3 -Mariana's reference site 1-3, MI1-MI3 -Mariana’s impacted sites 1-3, RR1-RR3 -Rio Casca’s Reference sites 1-3, RI1-RI3 -Rio Casca’s impacted sites 1-3, IR1-IR3 -Ipatinga’s Reference sites 1-3, II1-II3 -Ipatinga’s impacted sites 1-3, CR1-CR3 -Conselheiro Pena’s Reference sites 1-3, CI1-CI3 -Conselheiro Pena’s impacted sites 1-3, AR1-AR3 -Aimores’s Reference sites 1-3, AI1-AI3 -Aimores’s impacted sites 1-3. Ric. - richness.

Table S5. Mean biomass (fresh weight in g/m^2^) per earthworm species collected in five riparian forest regions: Mariana (M), Rio Casca (R), Ipatinga (I), Conselheiro Pena (C) and Aimorés (A). In each region, 15 non-impacted sites (reference, R, n=15) and 15 impacted sites (I, n=15) were selected per region. The numbers (1, 2, 3) refer to the three replicate sites in each region.

| Site | Mean biomass (fresh weight in g/m^2^) | | | | | | | |
| --- | --- | --- | --- | --- | --- | --- | --- | --- |
|  | Invasive earthworms | | Native earthworms | | | | | |
|  | *Pontoscolex* | *Amynthas* | *Rhinodrilus* | | *Righiodrilus* | | | Ocnerodrilidae |
|  | *P. corenthurus* | *A. gracilis* | *Rhinodrilus* sp.1 | *R. motucu* | *Righiodrilus* sp.1 | *Righiodrilus* sp.2 | *Righiodrilus* sp.3 | Ocnerodrilidae sp. |
| MR1 | 21.38 | 1.78 | 0.00 | 0.00 | 0.00 | 0.00 | 0.00 | 0.00 |
| MR2 | 25.00 | 0.00 | 0.00 | 0.00 | 0.00 | 0.12 | 0.00 | 0.00 |
| MR3 | 7.17 | 0.00 | 0.00 | 0.00 | 0.00 | 0.00 | 0.00 | 0.00 |
| MI1 | 8.23 | 0.00 | 0.00 | 0.00 | 0.00 | 0.00 | 0.00 | 0.00 |
| MI2 | 27.27 | 0.72 | 0.00 | 0.00 | 0.05 | 0.08 | 0.00 | 0.00 |
| MI3 | 19.18 | 0.00 | 0.00 | 0.00 | 0.00 | 0.10 | 0.00 | 0.00 |
| RR1 | 0.03 | 0.00 | 0.00 | 0.00 | 0.00 | 0.00 | 0.00 | 0.00 |
| RR2 | 6.72 | 0.00 | 0.00 | 0.00 | 0.00 | 0.00 | 0.00 | 0.00 |
| RR3 | 11.88 | 0.00 | 0.00 | 11.03 | 0.00 | 0.00 | 0.00 | 0.00 |
| RI1 | 2.08 | 0.00 | 0.00 | 0.00 | 0.03 | 0.00 | 0.00 | 0.00 |
| RI2 | 5.28 | 0.75 | 0.00 | 0.00 | 0.00 | 0.00 | 0.00 | 0.00 |
| RI3 | 3.48 | 0.00 | 0.00 | 0.00 | 0.10 | 0.00 | 0.00 | 0.00 |
| IR1 | 3.05 | 0.00 | 0.00 | 0.00 | 0.00 | 0.00 | 0.00 | 0.00 |
| IR2 | 2.78 | 0.00 | 0.00 | 0.00 | 0.00 | 0.00 | 0.00 | 0.00 |
| IR3 | 9.62 | 0.48 | 0.00 | 0.00 | 0.00 | 0.00 | 0.05 | 0.00 |
| II1 | 15.63 | 0.00 | 0.00 | 0.00 | 0.00 | 0.00 | 0.00 | 0.08 |
| II2 | 3.90 | 1.58 | 0.00 | 0.00 | 0.00 | 0.00 | 0.00 | 0.00 |
| II3 | 4.20 | 0.00 | 0.00 | 0.00 | 0.00 | 0.00 | 0.00 | 0.00 |
| CR1 | 0.07 | 0.00 | 0.00 | 0.00 | 0.00 | 0.00 | 0.00 | 0.00 |
| CR2 | 0.00 | 0.00 | 9.10 | 0.00 | 0.15 | 0.00 | 0.00 | 0.00 |
| CR3 | 0.07 | 0.00 | 10.25 | 0.00 | 0.00 | 0.00 | 0.00 | 0.00 |
| CI1 | 12.80 | 0.00 | 0.03 | 0.00 | 0.00 | 0.00 | 0.00 | 0.00 |
| CI2 | 0.00 | 0.00 | 2.82 | 0.00 | 0.00 | 0.00 | 0.00 | 0.00 |
| CI3 | 0.00 | 0.00 | 0.00 | 0.00 | 0.00 | 0.00 | 0.00 | 0.00 |
| AR1 | 0.00 | 0.00 | 1.17 | 0.00 | 0.00 | 0.00 | 0.00 | 0.00 |
| AR2 | 0.00 | 0.00 | 0.00 | 0.00 | 0.00 | 0.00 | 0.00 | 0.00 |
| AR3 | 0.87 | 0.00 | 0.00 | 0.00 | 0.00 | 0.00 | 0.00 | 0.00 |
| AI1 | 3.92 | 0.00 | 0.00 | 0.00 | 0.00 | 0.00 | 0.00 | 0.00 |
| AI2 | 0.00 | 0.00 | 0.00 | 0.00 | 0.00 | 0.00 | 0.00 | 0.00 |
| AI3 | 0.92 | 0.00 | 0.00 | 0.00 | 0.00 | 0.00 | 0.00 | 0.00 |

*Note*: Designations: M -Mariana, R -Rio Casca, I -Ipatinga, C -Conselheiro Pena, A -Aimores, I -Impacted sites, R -Reference sites, MR1-MR3 -Mariana's reference site 1-3, MI1-MI3 -Mariana’s impacted sites 1-3, RR1-RR3 -Rio Casca’s Reference sites 1-3, RI1-RI3 -Rio Casca’s impacted sites 1-3, IR1-IR3 -Ipatinga’s Reference sites 1-3, II1-II3 -Ipatinga’s impacted sites 1-3, CR1-CR3 -Conselheiro Pena’s Reference sites 1-3, CI1-CI3 -Conselheiro Pena’s impacted sites 1-3, AR1-AR3 -Aimores’s Reference sites 1-3, AI1-AI3 -Aimores’s impacted sites 1-3.

Table S6. Results of Generalized linear mixed models constructed to evaluate the effects of the native forest cover and soil attributes on total, native and invasive earthworm species abundance between reference sites and impacted sites. The interaction between reference and impacted sites and environmental variables were used as explanatory variables. Sites and regions were considered as random effects to be controlled in each model. The models were subjected to analysis of variance (ANOVA) tests. P-values < 0.05 highlighted in bold.

| **Total abundance of Earthworms (m^-2^)** | | | | | | | | | | | | | | | | |
| --- | --- | --- | --- | --- | --- | --- | --- | --- | --- | --- | --- | --- | --- | --- | --- | --- |
| **Explanatory variables** | **Chisq** | | | **Df** | | | | |  | | **P-Value** |  | |  | |  |
| Sites | 1.3947 | | | 1 | | | | |  | | 0.2376 |  | |  | |  |
| **pH** | **9741.60** | | | **1** | | | | | **<** | | **0.001** | Conditional | | R2: | | 1.00 |
| **pH:Sites** | **5080.90** | | | **1** | | | | | **<** | | **0.001** | Marginal | | R2: | | 0.47 |
| **P** | **498.31** | | | **1** | | | | | **<** | | **0.001** | Conditional | | R2: | | 1.00 |
| **P:Sites** | **232.67** | | | **1** | | | | | **<** | | **0.001** | Marginal | | R2: | | 0.00 |
| **K** | **127.59** | | | **1** | | | | | **<** | | **0.001** | Conditional | | R2: | | 1.00 |
| **K:Sites** | **3677.12** | | | **1** | | | | | **<** | | **0.001** | Marginal | | R2: | | 0.05 |
| **Ca** | **11041.6** | | | **1** | | | | | **<** | | **0.001** | Conditional | | R2: | | 1.00 |
| **Ca:Sites** | **5670.60** | | | **1** | | | | | **<** | | **0.001** | Marginal | | R2: | | 0.27 |
| **Mg** | **2490.20** | | | **1** | | | | | **<** | | **0.001** | Conditional | | R2: | | 1.00 |
| **Mg:Sites** | **5067.40** | | | **1** | | | | | **<** | | **0.001** | Marginal | | R2: | | 0.12 |
| **Al** | **2872.93** | | | **1** | | | | | **<** | | **0.001** | Conditional | | R2: | | 1.00 |
| **Al:Sites** | **409.96** | | | **1** | | | | | **<** | | **0.001** | Marginal | | R2: | | 0.01 |
| **Effective CEC** | **10898.7** | | | **1** | | | | | **<** | | **0.001** | Conditional | | R2: | | 1.00 |
| **Effective CEC:Sites** | **4452.9** | | | **1** | | | | | **<** | | **0.001** | Marginal | | R2: | | 0.26 |
| **Bsat** | **15077.10** | | | **1** | | | | | **<** | | **0.001** | Conditional | | R2: | | 1.00 |
| **Bsat:Sites** | **4065.70** | | | **1** | | | | | **<** | | **0.001** | Marginal | | R2: | | 0.23 |
| **C** | **1539.60** | | | **1** | | | | | **<** | | **0.001** | Conditional | | R2: | | 1.00 |
| **C:Sites** | **2696.60** | | | **1** | | | | | **<** | | **0.001** | Marginal | | R2: | | 0.03 |
| **S** | **35.481** | | | **1** | | | | | **<** | | **0.001** | Conditional | | R2: | | 1.00 |
| **S:Sites** | **328.177** | | | **1** | | | | | **<** | | **0.001** | Marginal | | R2: | | 0.00 |
| **Cu** | **48.391** | | | **1** | | | | | **<** | | **0.001** | Conditional | | R2: | | 1.00 |
| **Cu:Sites** | **20.041** | | | **1** | | | | |  | | **0.0000076** | Marginal | | R2: | | 0.00 |
| **Mn** | **252** | | | **1** | | | | | **<** | | **0.001** | Conditional | | R2: | | 1.00 |
| **Mn:Sites** | **1904.2** | | | **1** | | | | | **<** | | **0.001** | Marginal | | R2: | | 0.02 |
| Fe | 1.2314 | | | 1 | | | | |  | | 0.267 | Conditional | | R2: | | 1.00 |
| **Fe:Sites** | **13.082** | | | **1** | | | | |  | | **0.0003** | Marginal | | R2: | | 0 |
| **Zn** | **3023.6** | | | **1** | | | | | **<** | | **0.001** | Conditional | | R2: | | 1.00 |
| **Zn:Sites** | **4682** | | | **1** | | | | | **<** | | **0.001** | Marginal | | R2: | | 0.07 |
| **Cs** | **75.38** | | | **1** | | | | | **<** | | **0.001** | Conditional | | R2: | | 1.00 |
| **Cs:Sites** | **552.21** | | | **1** | | | | | **<** | | **0.001** | Marginal | | R2: | | 0.01 |
| Fs | 2.2909 | | | 1 | | | | |  | | 0.1301 | Conditional | | R2: | | 1.00 |
| **Fs:Sites** | **112.476** | | | **1** | | | | | **<** | | **0.001** | Marginal | | R2: | | 0.00 |
| **Silt** | **2084.1** | | | **1** | | | | | **<** | | **0.001** | Conditional | | R2: | | 1.00 |
| **Silt:Sites** | **20.411** | | | **1** | | | | |  | | **0.000006** | Marginal | | R2: | | 0.01 |
| **Clay** | **4319.83** | | | **1** | | | | | **<** | | **0.001** | Conditional | | R2: | | 1.00 |
| **Clay:Sites** | **233.89** | | | **1** | | | | | **<** | | **0.001** | Marginal | | R2: | | 0.04 |
| **DP** | **684.37** | | | **1** | | | | | **<** | | **0.001** | Conditional | | R2: | | 1 |
| **DP:Sites** | **4527.25** | | | **1** | | | | | **<** | | **0.001** | Marginal | | R2: | | 0.52 |
| Forest cover | 4.3883 | | | 1 | | | | |  | | 0.06099 | Conditional | | R2: | | 0.35 |
| Forest cover:Sites | 0.0548 | | | 1 | | | | |  | | 0.81678 | Marginal | | R2: | | 0.23 |
| **Abundance of Invasive Earthworms(m^-2^)** | | | | | | | | | | | | | | | | |
| **Explanatory variables** | | **Chisq** | | | | **Df** | |  | | **P-Value** | |  |  | |  | |
| Sites | | 0.2943 | | | | 1 | |  | | 0.5875 | |  |  | |  | |
| **pH** | | **170.95** | | | | **1** | | **<** | | **0.001** | | Conditional | R2: | | 1.00 | |
| **pH:Sites** | | **208.64** | | | | **1** | | **<** | | **0.001** | | Marginal | R2: | | 0.48 | |
| P | | 0.0229 | | | | 1 | |  | | 0.88 | | Conditional | R2: | | 0.96 | |
| **P:Sites** | | **6.9325** | | | | **1** | |  | | **0.0085** | | Marginal | R2: | | 0.01 | |
| K | | 3.571 | | | | 1 | |  | | 0.0588 | | Conditional | R2: | | 0.96 | |
| **K:Sites** | | **23.882** | | | | **1** | |  | | **0.0000010** | | Marginal | R2: | | 0.04 | |
| **Ca** | | **133** | | | | **1** | | **<** | | **0.001** | | Conditional | R2: | | 0.98 | |
| **Ca:Sites** | | **224.68** | | | | **1** | | **<** | | **0.001** | | Marginal | R2: | | 0.25 | |
| **Mg** | | **45.678** | | | | **1** | | **<** | | **0.001** | | Conditional | R2: | | 0.96 | |
| **Mg:Sites** | | **36.692** | | | | **1** | | **<** | | **0.001** | | Marginal | R2: | | 0.11 | |
| **Al** | | **103.093** | | | | **1** | | **<** | | **0.001** | | Conditional | R2: | | 0.96 | |
| Al:Sites | | 3.1626 | | | | 1 | |  | | 0.0753 | | Marginal | R2: | | 0.17 | |
| **Effective CEC** | | **118.44** | | | | **1** | | **<** | | **0.001** | | Conditional | R2: | | 0.98 | |
| **Effective CEC:Sites** | | **133.53** | | | | **1** | | **<** | | **0.001** | | Marginal | R2: | | 0.27 | |
| **Bsat** | | **321.91** | | | | **1** | | **<** | | **0.001** | | Conditional | R2: | | 0.98 | |
| **Bsat:Sites** | | **187.23** | | | | **1** | | **<** | | **0.001** | | Marginal | R2: | | 0.39 | |
| **C** | | **36.159** | | | | **1** | | **<** | | **0.001** | | Conditional | R2: | | 0.96 | |
| **C:Sites** | | **45.191** | | | | **1** | | **<** | | **0.0019** | | Marginal | R2: | | 0.06 | |
| S | | 0.9128 | | | | 1 | |  | | 0.3393 | | Conditional | R2: | | 0.96 | |
| **S:Sites** | | **7.9939** | | | | **1** | |  | | **0.00469** | | Marginal | R2: | | 0.00 | |
| **Cu** | | **14.915** | | | | **1** | |  | | **0.00011** | | Conditional | R2: | | 0.96 | |
| **Cu:Sites** | | **34.76** | | | | **1** | | **<** | | **0.0019** | | Marginal | R2: | | 0.08 | |
| Mg | | 2.6307 | | | | 1 | |  | | 0.1048 | | Conditional | R2: | | 0.96 | |
| Mg:Sites | | 0.214 | | | | 1 | |  | | 0.6436 | | Marginal | R2: | | 0.00 | |
| **Fe** | | **33.28** | | | | **1** | | **<** | | **0.001** | | Conditional | R2: | | 0.96 | |
| **Fe:Sites** | | **31.069** | | | | **1** | | **<** | | **0.001** | | Marginal | R2: | | 0.04 | |
| Zn | | 0.740 | | | | 1 | |  | | 0.3895 | | Conditional | R2: | | 0.96 | |
| **Zn:Sites** | | **52.377** | | | | **1** | | **<** | | **0.001** | | Marginal | R2: | | 0.07 | |
| **Cs** | | **11.458** | | | | **1** | |  | | **0.00071** | | Conditional | R2: | | 0.95 | |
| **Cs:Sites** | | **13.525** | | | | **1** | |  | | **0.00024** | | Marginal | R2: | | 0.04 | |
| **Fs** | | **17.502** | | | | **1** | |  | | **0.00003** | | Conditional | R2: | | 0.96 | |
| Fs:Sites | | 2.622 | | | | 1 | |  | | 0.1054 | | Marginal | R2: | | 0.02 | |
| Silt | | 0.056 | | | | 1 | | < | | **0.001** | | Conditional | R2: | | 0.96 | |
| Silt:Sites | | 0.717 | | | | 1 | |  | | 0.397 | | Marginal | R2: | | 0.00 | |
| **Clay** | | **125.396** | | | | **1** | | **<** | | **0.001** | | Conditional | R2: | | 0.97 | |
| **Clay:Sites** | | **49.008** | | | | **1** | | **<** | | **0.001** | | Marginal | R2: | | 0.25 | |
| **DP** | | **0.0854** | | | | **1** | |  | | 0.770 | | Conditional | R2: | | 1.00 | |
| **DP:Sites** | | **60.497** | | | | **1** | | **<** | | **0.001** | | Marginal | R2: | | 0.50 | |
| **Forest cover** | | **5.1081** | | | | **1** | |  | | **0.0473** | | Conditional | R2: | | 0.36 | |
| Forest cover:Sites | | 0.0262 | | | | 1 | |  | | 0.8727 | | Marginal | R2: | | 0.25 | |
| **Abundance of Native Earthworms(m^-2^)** | | | | | | | | | | | | | | | | |
| **Explanatory variables** | | | **Chisq** | | **Df** | | **P-Value** | | | | |  |  | |  | |
| Sites | | | 1.964 | | 1 | | 0.161 | | | | |  |  | |  | |
| pH | | | 1.119 | | 1 | | 0.290 | | | | | Conditional | R2: | | 0.54 | |
| pH:Sites | | | 1.312 | | 1 | | 0.252 | | | | | Marginal | R2: | | 0.13 | |
| P | | | 2.882 | | 1 | | 0.090 | | | | | Conditional | R2: | | 0.66 | |
| P:Sites | | | 0.0002 | | 1 | | 0.989 | | | | | Marginal | R2: | | 0.1 | |
| K | | | 2.362 | | 1 | | 0.124 | | | | | Conditional | R2: | | 0.44 | |
| K:Sites | | | 1.711 | | 1 | | 0.191 | | | | | Marginal | R2: | | 0.17 | |
| Ca | | | 0.009 | | 1 | | 0.929 | | | | | Conditional | R2: | | 0.57 | |
| Ca:Sites | | | 0.007 | | 1 | | 0.934 | | | | | Marginal | R2: | | 0.00 | |
| Mg | | | 3.018 | | 1 | | 0.082 | | | | | Conditional | R2: | | 0.57 | |
| Mg:Sites | | | 0.560 | | 1 | | 0.454 | | | | | Marginal | R2: | | 0.14 | |
| Al | | | 0.211 | | 1 | | 0.646 | | | | | Conditional | R2: | | 0.58 | |
| **Al:Sites** | | | **13.676** | | **1** | | **0.0002** | | | | | Marginal | R2: | | 0.26 | |
| Effective CEC | | | 0.308 | | 1 | | 0.579 | | | | | Conditional | R2: | | 0.57 | |
| Effective CEC:Sites | | | 0.094 | | 1 | | 0.759 | | | | | Marginal | R2: | | 0.02 | |
| Bsat | | | 0.038 | | 1 | | 0.846 | | | | | Conditional | R2: | | 0.55 | |
| Bsat:Sites | | | 0.216 | | 1 | | 0.642 | | | | | Marginal | R2: | | 0.02 | |
| **C** | | | **5.061** | | **1** | | **0.025** | | | | | Conditional | R2: | | 0.63 | |
| C:Sites | | | 3.265 | | 1 | | 0.071 | | | | | Marginal | R2: | | 0.29 | |
| S | | | 0.051 | | 1 | | 0.820 | | | | | Conditional | R2: | | 0.61 | |
| **S:Sites** | | | **8.821** | | **1** | | **0.003** | | | | | Marginal | R2: | | 0.32 | |
| **Cu** | | | **4.425** | | **1** | | **0.035** | | | | | Conditional | R2: | | 0.69 | |
| Cu:Sites | | | 0.139 | | 1 | | 0.709 | | | | | Marginal | R2: | | 0.23 | |
| Mg | | | 1.681 | | 1 | | 0.195 | | | | | Conditional | R2: | | 0.58 | |
| Mg:Sites | | | 1.094 | | 1 | | 0.296 | | | | | Marginal | R2: | | 0.13 | |
| Fe | | | 0.280 | | 1 | | 0.597 | | | | | Conditional | R2: | | 0.64 | |
| Fe:Sites | | | 0.887 | | 1 | | 0.346 | | | | | Marginal | R2: | | 0.04 | |
| **Zn** | | | **4.055** | | **1** | | **0.044** | | | | | Conditional | R2: | | 0.52 | |
| **Zn:Sites** | | | **5.256** | | **1** | | **0.022** | | | | | Marginal | R2: | | 0.29 | |
| **Cs** | | | **6.538** | | **1** | | **0.0106** | | | | | Conditional | R2: | | 0.69 | |
| Cs:Sites | | | 0.729 | | 1 | | 0.393 | | | | | Marginal | R2: | | 0.17 | |
| Fs | | | 0.388 | | 1 | | 0.534 | | | | | Conditional | R2: | | 0.54 | |
| Fs:Sites | | | 0.295 | | 1 | | 0.587 | | | | | Marginal | R2: | | 0.02 | |
| Silt | | | 1.519 | | 1 | | 0.218 | | | | | Conditional | R2: | | 0.62 | |
| Silt:Sites | | | 1.079 | | 1 | | 0.299 | | | | | Marginal | R2: | | 0.12 | |
| **Clay** | | | **6.245** | | **1** | | **0.013** | | | | | Conditional | R2: | | 0.66 | |
| Clay:Sites | | | 2.047 | | 1 | | 0.153 | | | | | Marginal | R2: | | 0.28 | |
| DP | | | 1.434 | | 1 | | 0.231 | | | | | Conditional | R2: | | 0.62 | |
| DP:Sites | | | 3.259 | | 1 | | 0.071 | | | | | Marginal | R2: | | 0.21 | |
| Forest cover | | | 0.463 | | 1 | | 7.968 | | | | | Conditional | R2: | | 0.10 | |
| Forest cover:Sites | | | 0.0019 | | 1 | | 24.563 | | | | | Marginal | R2: | | 0.03 | |

| **Total biomass of Earthworms (g.m-2)** | | | | | | | |
| --- | --- | --- | --- | --- | --- | --- | --- |
| **Explanatory variables** | **F** | **Df** | **Df.res** | **P-Value** |  |  |  |
| Sites | 0.0093 | 1 | 22.995 | 0.924 |  |  |  |
| pH | 0.2188 | 1 | 94.775 | 0.641 | Conditional | R2: | 0.22 |
| pH:Sites | 0.0061 | 1 | 24.446 | 0.938 | Marginal | R2: | 0.00 |
| P | 2.3124 | 1 | 126.375 | 0.131 | Conditional | R2: | 0.19 |
| P:Sites | 0.677 | 1 | 48.997 | 0.415 | Marginal | R2: | 0.02 |
| K | 1.026 | 1 | 137.53 | 0.313 | Conditional | R2: | 0.23 |
| K:Sites | 0.526 | 1 | 36.47 | 0.473 | Marginal | R2: | 0.01 |
| Ca | 0.024 | 1 | 64.017 | 0.877 | Conditional | R2: | 0.22 |
| Ca:Sites | 1.624 | 1 | 38.635 | 0.210 | Marginal | R2: | 0.02 |
| Mg | 0.1392 | 1 | 84.914 | 0.710 | Conditional | R2: | 0.22 |
| Mg:Sites | 1.1112 | 1 | 36.092 | 0.299 | Marginal | R2: | 0.01 |
| Al | 0.015 | 1 | 42.690 | 0.904 | Conditional | R2: | 0.22 |
| Al:Sites | 0.553 | 1 | 166.65 | 0.458 | Marginal | R2: | 0.01 |
| Effective CEC | 0.115 | 1 | 63.450 | 0.736 | Conditional | R2: | 0.22 |
| Effective CEC:Sites | 1.479 | 1 | 30.497 | 0.233 | Marginal | R2: | 0.02 |
| Bsat | 2.010 | 1 | 62.477 | 0.161 | Conditional | R2: | 0.25 |
| Bsat:Sites | 0.182 | 1 | 29.574 | 0.673 | Marginal | R2: | 0.03 |
| C | 0.0018 | 1 | 77.480 | 0.966 | Conditional | R2: | 0.22 |
| C:Sites | 0.040 | 1 | 47.819 | 0.842 | Marginal | R2: | 0.00 |
| S | 0.448 | 1 | 167.44 | 0.504 | Conditional | R2: | 0.21 |
| S:Sites | 0.060 | 1 | 108.84 | 0.810 | Marginal | R2: | 0.00 |
| Cu | 2.292 | 1 | 107.802 | 0.133 | Conditional | R2: | 0.20 |
| Cu:Sites | 0.004 | 1 | 35.334 | 0.951 | Marginal | R2: | 0.02 |
| Mn | 1.3116 | 1 | 137.169 | 0.254 | Conditional | R2: | 0.23 |
| Mn:Sites | 0.5168 | 1 | 44.921 | 0.476 | Marginal | R2: | 0.02 |
| Fe | 0.444 | 1 | 166.569 | 0.506 | Conditional | R2: | 0.25 |
| Fe:Sites | 0.324 | 1 | 70.081 | 0.571 | Marginal | R2: | 0.01 |
| Zn | 0.3445 | 1 | 157.04 | 0.558 | Conditional | R2: | 0.22 |
| Zn:Sites | 1.391 | 1 | 44.52 | 0.245 | Marginal | R2: | 0.02 |
| Cs | 0.972 | 1 | 130.739 | 0.326 | Conditional | R2: | 0.21 |
| Cs:Sites | 0.174 | 1 | 59.968 | 0.678 | Marginal | R2: | 0.01 |
| Fs | 0.614 | 1 | 125.637 | 0.435 | Conditional | R2: | 0.23 |
| Fs:Sites | 0.036 | 1 | 42.836 | 0.851 | Marginal | R2: | 0.01 |
| **Silt** | **6.363** | **1** | **85.187** | **0.014** | Conditional | R2: | 0.17 |
| Silt:Sites | 0.042 | 1 | 25.366 | 0.840 | Marginal | R2: | 0.05 |
| Clay | 0.472 | 1 | 81.118 | 0.494 | Conditional | R2: | 0.24 |
| Clay:Sites | 0.106 | 1 | 32.288 | 0.747 | Marginal | R2: | 0.01 |
| DP | 2.117 | 1 | 69.81 | 0.150 | Conditional | R2: | 0.22 |
| DP:Sites | 0.014 | 1 | 24.75 | 0.906 | Marginal | R2: | 0.02 |
| Forest cover | 0.059 | 1 | 10.26 | 0.814 | Conditional | R2: | 0.27 |
| Forest cover:Sites | 0.935 | 1 | 21.19 | 0.345 | Marginal | R2: | 0.04 |

| **Biomass of Invasive Earthworms (g.m-2)** | | | | | | | | |
| --- | --- | --- | --- | --- | --- | --- | --- | --- |
| **Explanatory variables** | **F** | **Df** | **Df.res** | **P-Value** |  | |  |  |
| Sites | 0.4065 | 1 | 20.683 | 0.531 |  | |  |  |
| pH | 0.0183 | 1 | 87.948 | 0.893 | Conditional | R2: | 0.24 | |
| pH:Sites | 0.8389 | 1 | 24.137 | 0.369 | Marginal | R2: | 0.01 | |
| P | 1.1117 | 1 | 114.371 | 0.294 | Conditional | R2: | 0.19 | |
| P:Sites | 0.7128 | 1 | 42.217 | 0.403 | Marginal | R2: | 0.02 | |
| K | 1.877 | 1 | 131.843 | 0.173 | Conditional | R2: | 0.21 | |
| K:Sites | 0.0015 | 1 | 35.174 | 0.969 | Marginal | R2: | 0.01 | |
| **Ca** | **5.7855** | **1** | **99.119** | **0.018** | Conditional | R2: | 0.23 | |
| Ca:Sites | 0.1884 | 1 | 38.632 | 0.667 | Marginal | R2: | 0.05 | |
| Mg | 2.1299 | 1 | 73.136 | 0.149 | Conditional | R2: | 0.21 | |
| Mg:Sites | 0.0026 | 1 | 32.156 | 0.960 | Marginal | R2: | 0.02 | |
| Al | 0.0006 | 1 | 38.461 | 0.980 | Conditional | R2: | 0.22 | |
| Al:Sites | 0.351 | 1 | 148.78 | 0.555 | Marginal | R2: | 0.00 | |
| Effective CEC | 7.6959 | 1 | 74.50 | 0.007 | Conditional | R2: | 0.21 | |
| Effective CEC:Sites | 0.117 | 1 | 27.823 | 0.735 | Marginal | R2: | 0.07 | |
| Bsat | 4.605 | 1 | 54.947 | 0.036 | Conditional | R2: | 0.27 | |
| Bsat:Sites | 0.9481 | 1 | 31.218 | 0.338 | Marginal | R2: | 0.06 | |
| C | 0.3524 | 1 | 64.255 | 0.555 | Conditional | R2: | 0.22 | |
| C:Sites | 0.0425 | 1 | 42.424 | 0.838 | Marginal | R2: | 0.01 | |
| S | 0.171 | 1 | 151.49 | 0.680 | Conditional | R2: | 0.22 | |
| S:Sites | 0.024 | 1 | 98.004 | 0.878 | Marginal | R2: | 0.00 | |
| Cu | 1.096 | 1 | 108.38 | 0.298 | Conditional | R2: | 0.20 | |
| Cu:Sites | 0.154 | 1 | 32.41 | 0.698 | Marginal | R2: | 0.01 | |
| Mn | 2.558 | 1 | 115.761 | 0.113 | Conditional | R2: | 0.22 | |
| Mn:Sites | 0.0057 | 1 | 37.323 | 0.940 | Marginal | R2: | 0.02 | |
| Fe | 2.016 | 1 | 152.196 | 0.158 | Conditional | R2: | 0.27 | |
| Fe:Sites | 0.416 | 1 | 61.156 | 0.522 | Marginal | R2: | 0.02 | |
| Zn | 2.698 | 1 | 131.245 | 0.103 | Conditional | R2: | 0.20 | |
| Zn:Sites | 0.011 | 1 | 34.925 | 0.919 | Marginal | R2: | 0.02 | |
| Cs | 2.191 | 1 | 119.599 | 0.141 | Conditional | R2: | 0.20 | |
| Cs:Sites | 0.102 | 1 | 51.723 | 0.750 | Marginal | R2: | 0.02 | |
| Fs | 0.294 | 1 | 102.86 | 0.589 | Conditional | R2: | 0.23 | |
| Fs:Sites | 0.389 | 1 | 36.8 | 0.537 | Marginal | R2: | 0.01 | |
| **Silt** | **20.06** | **1** | **47.51** | **< 0.001** | Conditional | R2: | 0.17 | |
| Silt:Sites | 1.190 | 1 | 15.883 | 0.292 | Marginal | R2: | 0.14 | |
| Clay | 0.026 | 1 | 59.736 | 0.872 | Conditional | R2: | 0.23 | |
| Clay:Sites | 0.075 | 1 | 25.387 | 0.787 | Marginal | R2: | 0.00 | |
| DP | 5.116 | 1 | 47.673 | 0.028 | Conditional | R2: | 0.20 | |
| DP:Sites | 0.852 | 1 | 22.189 | 0.366 | Marginal | R2: | 0.07 | |
| Forest cover | 0.902 | 1 | 11.452 | 0.362 | Conditional | R2: | 0.37 | |
| Forest cover:Sites | 2.307 | 1 | 19.056 | 0.145 | Marginal | R2: | 0.17 | |

| **Biomass of Native Earthworms (g.m-2)** | | | | | | | |
| --- | --- | --- | --- | --- | --- | --- | --- |
| **Explanatory variables** | **F** | **Df** | **Df.res** | **P-Value** |  |  |  |
| **Sites** | **5.0443** | **1** | **10.198** | **0.048** |  |  |  |
| pH | 0.8381 | 1 | 13.410 | 0.376 | Conditional | R2: | 0.27 |
| **pH:Sites** | **5.2231** | **1** | **9.828** | **0.046** | Marginal | R2: | 0.23 |
| P | 1.1118 | 1 | 17.801 | 0.306 | Conditional | R2: | 0.19 |
| P:Sites | 0.6638 | 1 | 17.126 | 0.426 | Marginal | R2: | 0.08 |
| K | 0.8675 | 1 | 16.374 | 0.365 | Conditional | R2: | 0.25 |
| K:Sites | 5.1334 | 1 | 7.449 | 0.056 | Marginal | R2: | 0.24 |
| Ca | 5.6817 | 1 | 8.225 | 0.044 | Conditional | R2: | 0.52 |
| Ca:Sites | 1.8703 | 1 | 11.377 | 0.198 | Marginal | R2: | 0.36 |
| **Mg** | **8.4464** | **1** | **11.231** | **0.014** | Conditional | R2: | NA |
| Mg:Sites | 4.2177 | 1 | 10.223 | 0.067 | Marginal | R2: | 0.38 |
| Al | 0.6135 | 1 | 13.402 | 0.447 | Conditional | R2: | 0.22 |
| Al:Sites | 0.0032 | 1 | 22.977 | 0.956 | Marginal | R2: | 0.03 |
| **Effective CEC** | **7.0028** | **1** | **8.9238** | **0.027** | Conditional | R2: | 0.53 |
| Effective CEC:Sites | 2.0276 | 1 | 10.427 | 0.184 | Marginal | R2: | 0.39 |
| Bsat | 2.8032 | 1 | 8.903 | 0.129 | Conditional | R2: | 0.37 |
| **Bsat:Sites** | **5.9** | **1** | **9.520** | **0.037** | Marginal | R2: | 0.32 |
| **C** | **7.4374** | **1** | **19.649** | **0.013** | Conditional | R2: | NA |
| C:Sites | 4.5852 | 1 | 11.304 | 0.055 | Marginal | R2: | 0.35 |
| S | 2.4746 | 1 | 14.767 | 0.137 | Conditional | R2: | 0.28 |
| S:Sites | 3.1628 | 1 | 11.421 | 0.102 | Marginal | R2: | 0.23 |
| Cu | 0.8522 | 1 | 10.325 | 0.377 | Conditional | R2: | 0.23 |
| Cu:Sites | 3.761 | 1 | 5.676 | 0.103 | Marginal | R2: | 0.178 |
| Mn | 3.958 | 1 | 17.922 | 0.062 | Conditional | R2: | NA |
| Mn:Sites | 1.727 | 1 | 20.596 | 0.203 | Marginal | R2: | 0.207 |
| Fe | 2.346 | 1 | 19.344 | 0.142 | Conditional | R2: | 0.22 |
| Fe:Sites | 0.261 | 1 | 20.860 | 0.615 | Marginal | R2: | 0.10 |
| Zn | 2.317 | 1 | 7.4315 | 0.169 | Conditional | R2: | NA |
| **Zn:Sites** | **6.725** | **1** | **11.520** | **0.024** | Marginal | R2: | 0.31 |
| Cs | 3.643 | 1 | 16.580 | 0.074 | Conditional | R2: | 0.23 |
| Cs:Sites | 1.360 | 1 | 18.210 | 0.259 | Marginal | R2: | 0.21 |
| Fs | 3.887 | 1 | 22.942 | 0.061 | Conditional | R2: | NA |
| Fs:Sites | 1.542 | 1 | 16.079 | 0.232 | Marginal | R2: | 0.19 |
| Silt | 2.610 | 1 | 13.364 | 0.130 | Conditional | R2: | 0.23 |
| Silt:Sites | 1.228 | 1 | 11.653 | 0.290 | Marginal | R2: | 0.16 |
| Clay | 4.184 | 1 | 19.693 | 0.054 | Conditional | R2: | NA |
| Clay:Sites | 5.491 | 1 | 11.010 | 0.039 | Marginal | R2: | 0.31 |
| PD | 3.470 | 1 | 12.328 | 0.087 | Conditional | R2: | NA |
| PD:Sites | 4.318 | 1 | 6.878 | 0.077 | Marginal | R2: | 0.28 |
| Forest cover | 1.020 | 1 | 1.957 | 0.421 | Conditional | R2: | NA |
| Forest cover:Sites | 1.660 | 1 | 8.871 | 0.230 | Marginal | R2: | 0.26 |

*Note*: Designations: RS -reference sites, IS -impacted sites, GLMM -Generalized linear mixed models, ANOVA -analysis of variance. Soil properties: P -phosphorus (mg/dm^3^), K -potassium (cmolc/dm^3^), Ca -calcium (cmolc/dm^3^), Mg -magnesium ( (cmolc/dm^3^), Al -aluminum ( (cmolc/dm^3^), C -carbon (g/kg), S -sulfur (mg/dm^3^), Cu -copper ((mg/dm^3^), Mn -manganese (mg/dm^3^), Fe -iron ((mg/dm^3^), Zn -zinc ((mg/dm^3^), Bsat -base saturation (%), Cs -coarse sand (kg/kg), Fs -fine sand (kg/kg), Silt (kg/kg), Clay (Kg/kg), PD - Particle density (g/cm^3^).

Table S7. Equations of the models tested (y= ax+b), where "a" is the slope and "b" is the intercept. Values in bold correspond to significant tests (p< 0.05).

|  |  | | **Total abundance** | | **Invasive abundance** | | **Native abundance** | | **Total biomass** | | **Invasive biomass** | | **Native biomass** | |
| --- | --- | --- | --- | --- | --- | --- | --- | --- | --- | --- | --- | --- | --- | --- |
|  |  |  | a | b | a | b | a | b | a | b | a | b | a | b |
| *Plant cover* | | | | | | | | | | | | | | |
| Forest cover - 1000m | General | | 0.016 | 5.627 | 0.016 | 2.610 | 0.004 | 0.131 | 0.048 | 16.800 | 0.140 | 10.516 | -0.226 | 20.905 |
|  | Site | IS | 0.007 | 6.115 | 0.005 | 3.182 | 0.001 | 0.099 | -0.269 | 27.958 | -0.191 | 25.639 | -0.117 | 7.898 |
|  |  | RS | 0.027 | 1.040 | 0.063 | -0.957 | 0.055 | 1.257 | 0.005 | 16.720 | 0.420 | -6.192 | -0.369 | 38.926 |
| *Soil properties* | | | | | | | | | | | | | | |
| pH | General | | **-0.499** | **9.235** | **-0.329** | **5.947** | -0.094 | 3.924 | 1.724 | 11.155 | 0.573 | 15.793 | 7.839 | -26.282 |
|  | Site | IS | **-0.258** | **8.345** | **-0.040** | **4.509** | -0.380 | 5.597 | 4.566 | -4.577 | 8.643 | -25.613 | **-0.880** | **8.859** |
|  |  | RS | **-1.027** | **11.569** | **-0.878** | **8.642** | 0.038 | 3.117 | -0.882 | 24.732 | -7.343 | 52.899 | **12.765** | **-44.574** |
| P | General | | **-0.023** | **6.729** | -0.001 | 4.236 | -0.031 | 3.525 | -0.825 | 23.881 | -0.586 | 21.511 | -1.634 | 23.505 |
|  | Site | IS | **-0.015** | **7.063** | **0.005** | **4.265** | -0.071 | 3.911 | -0.736 | 24.174 | -0.709 | 24.567 | 0.190 | 2.728 |
|  |  | RS | **-0.056** | **6.471** | **-0.027** | **4.261** | 0.006 | 3.317 | -2.120 | 26.810 | -1.032 | 20.243 | -1.108 | 29.310 |
| K | General | | **0.273** | **6.581** | 0.245 | 4.190 | 0.761 | 3.243 | -24.536 | 24.385 | -34.903 | 24.538 | 51.954 | 6.600 |
|  | Site | IS | **-0.752** | **7.102** | **-0.168** | **4.323** | -0.947 | 3.683 | -44.109 | 27.497 | -50.683 | 28.952 | 11.492 | 1.455 |
|  |  | RS | **3.359** | **5.706** | **1.362** | **3.934** | 0.916 | 3.133 | 20.262 | 16.799 | 6.607 | 15.661 | 96.097 | 7.022 |

| Ca | General | | **-0.262** | **7.256** | **-0.153** | **4.577** | 0.002 | 3.395 | -0.211 | 20.636 | **-4.067** | **27.277** | 4.581 | -1.250 |
| --- | --- | --- | --- | --- | --- | --- | --- | --- | --- | --- | --- | --- | --- | --- |
|  | Site | IS | **-0.084** | **7.165** | **0.038** | **4.212** | -0.098 | 3.754 | -4.778 | 30.831 | -6.039 | 33.917 | 1.034 | 0.987 |
|  |  | RS | **-0.432** | **7.366** | **-0.340** | **4.948** | 0.015 | 3.250 | 1.118 | 17.501 | -3.842 | 24.212 | 4.917 | 2.314 |
| Mg | General | | **-0.258** | **6.905** | **-0.149** | **4.384** | 0.131 | 3.236 | -1.165 | 21.374 | -4.933 | 23.805 | **16.616** | **-3.907** |
|  | Site | IS | **-0.038** | **7.009** | **0.038** | **4.212** | -0.040 | 3.528 | -5.637 | 25.654 | -6.845 | 27.360 | 3.589 | 0.064 |
|  |  | RS | **-0.771** | **7.140** | **-0.340** | **4.948** | 0.166 | 3.076 | 4.671 | 15.168 | -1.937 | 18.721 | 17.717 | 1.084 |
|  | General | | **0.451** | **6.550** | **0.405** | **4.156** | 0.212 | 3.389 | -0.670 | 20.261 | 0.135 | 18.695 | -25.297 | 19.047 |
| Al | Site | IS | **0.642** | **6.934** | **0.315** | **4.279** | **3.185** | **3.395** | 10.924 | 19.242 | 7.631 | 20.068 | -8.176 | 4.106 |
|  |  | RS | **0.299** | **6.228** | **0.465** | **4.028** | **-0.187** | **3.344** | -2.159 | 21.106 | 0.649 | 16.400 | -45.493 | 31.329 |
| Effective CEC | General | | **-0.222** | **7.472** | **-0.117** | **4.657** | 0.011 | 3.347 | -0.357 | 21.510 | -3.702 | 31.797 | **4.117** | **-5.864** |
|  | Site | IS | **-0.066** | **7.208** | **0.019** | **4.231** | -0.057 | 3.715 | -3.481 | 32.247 | -4.290 | 35.314 | 0.830 | 0.476 |
|  |  | RS | **-0.337** | **7.654** | **-0.232** | **5.062** | 0.023 | 3.172 | 1.137 | 15.577 | -3.202 | 28.214 | 4.763 | -5.406 |
| Bsat | General | | **-0.021** | **7.755** | **-0.015** | **5.024** | -0.001 | 3.443 | -0.165 | 28.782 | -0.257 | 31.827 | 0.464 | -10.761 |
|  | Site | IS | **-0.012** | **7.682** | **-0.004** | **4.528** | -0.018 | 4.618 | -0.547 | 51.908 | -0.560 | 53.284 | **0.093** | **-1.873** |
|  |  | RS | **-0.035** | **7.958** | **-0.028** | **5.507** | 0.003 | 3.127 | -0.043 | 22.250 | -0.263 | 27.734 | **0.586** | **-9.726** |
| C | General | | **0.132** | **6.440** | **0.092** | **4.110** | **0.184** | **3.119** | 0.073 | 20.015 | -1.471 | 20.835 | **15.085** | **-6.029** |
|  | Site | IS | **0.382** | **6.557** | **0.238** | **4.068** | 0.219 | 3.216 | -0.901 | 20.763 | -1.651 | 22.083 | -2.527 | 6.919 |
|  |  | RS | **0.008** | **6.299** | **0.021** | **4.140** | 0.253 | 2.863 | 0.582 | 19.179 | -0.734 | 18.167 | 26.228 | -19.734 |

| S | General | | **-0.002** | **6.644** | -0.001 | 4.242 | -0.005 | 3.428 | -0.117 | 21.152 | -0.072 | 19.422 | -2.016 | 27.891 |
| --- | --- | --- | --- | --- | --- | --- | --- | --- | --- | --- | --- | --- | --- | --- |
|  | Site | IS | **0.006** | **6.932** | **-0.008** | **4.340** | **0.127** | **2.918** | -0.199 | 21.000 | -0.353 | 22.593 | -0.614 | 6.550 |
|  |  | RS | **-0.004** | **6.361** | **0.000** | **4.169** | **-0.013** | **3.397** | -0.110 | 21.664 | -0.020 | 17.067 | -2.552 | 40.807 |
| Cu | General | | **0.021** | **6.597** | **0.054** | **4.144** | **0.130** | **3.206** | -3.319 | 25.596 | -2.452 | 23.043 | -4.079 | 22.541 |
|  | Site | IS | **0.034** | **6.909** | **0.135** | **4.051** | 0.156 | 3.137 | -3.607 | 26.358 | -3.362 | 26.582 | -1.249 | 6.694 |
|  |  | RS | **0.007** | **6.304** | **-0.033** | **4.224** | 0.098 | 3.243 | -3.148 | 24.917 | -1.764 | 19.685 | 4.605 | 23.116 |
| Mn | General | | **-0.001** | **6.683** | 0.000 | 4.208 | 0.001 | 3.274 | 0.036 | 16.772 | 0.050 | 13.937 | -0.184 | 36.610 |
|  | Site | IS | **-0.003** | **7.268** | 0.000 | 4.286 | 0.002 | 3.164 | -0.017 | 21.526 | 0.005 | 19.977 | -0.023 | 7.077 |
|  |  | RS | **0.000** | **6.283** | 0.000 | 4.149 | 0.000 | 3.326 | 0.052 | 15.602 | 0.061 | 11.000 | -0.067 | 32.514 |
| Fe | General | | 0.000 | 6.627 | **0.001** | **4.170** | 0.000 | 3.370 | 0.012 | 18.601 | 0.029 | 14.929 | -0.069 | 24.978 |
|  | Site | IS | **0.000** | **6.962** | **0.001** | **4.185** | -0.001 | 3.587 | 0.023 | 16.104 | 0.035 | 14.791 | -0.019 | 7.735 |
|  |  | RS | **0.000** | **6.328** | **-0.001** | **4.257** | 0.002 | 3.232 | -0.027 | 22.661 | 0.000 | 16.807 | -0.133 | 34.874 |
| Zn | General | | **-0.106** | **6.885** | 0.005 | 4.221 | **0.039** | **3.276** | -0.800 | 22.138 | -2.734 | 25.367 | 3.425 | 6.147 |
|  | Site | IS | **0.008** | **6.948** | **0.055** | **4.162** | **0.121** | **3.064** | -4.010 | 29.841 | -4.152 | 30.673 | **-2.457** | **12.640** |
|  |  | RS | **-0.225** | **6.843** | **-0.072** | **4.337** | **0.028** | **3.239** | 1.136 | 17.480 | -1.286 | 19.792 | **4.310** | **14.485** |
| Coarse sand | General | | **-0.162** | **6.669** | **-0.281** | **4.296** | **-0.859** | **3.566** | -12.259 | 22.820 | -19.245 | 22.754 | 59.785 | 6.011 |
|  | Site | IS | **0.078** | **6.954** | **0.055** | **4.162** | -1.680 | 3.663 | -17.116 | 23.013 | -19.001 | 24.065 | 4.766 | 3.400 |
|  |  | RS | **-0.911** | **6.575** | **-0.072** | **4.337** | -0.256 | 3.399 | -7.350 | 22.171 | -17.220 | 20.712 | 40.274 | 16.398 |

| Fine sand | General | | -0.026 | 6.638 | **-0.329** | **4.354** | -0.132 | 3.444 | -8.779 | 23.112 | -6.394 | 21.063 | -35.879 | 29.759 |
| --- | --- | --- | --- | --- | --- | --- | --- | --- | --- | --- | --- | --- | --- | --- |
|  | Site | IS | **-0.180** | **7.040** | -0.229 | 4.391 | -0.583 | 3.707 | -6.953 | 22.628 | -8.404 | 23.957 | 3.709 | 2.474 |
|  |  | RS | **0.184** | **6.264** | -0.467 | 4.328 | -0.211 | 3.381 | -11.403 | 23.443 | -8.562 | 19.341 | -34.316 | 36.523 |
| Silt | General | | **-1.050** | **6.813** | -0.026 | 4.237 | 0.685 | 3.257 | **45.998** | **11.431** | **73.465** | **4.540** | -68.497 | 31.660 |
|  | Site | IS | **-0.978** | **7.156** | 0.035 | 4.289 | 1.289 | 3.151 | 70.435 | 5.500 | 92.366 | 1.926 | -9.669 | 6.365 |
|  |  | RS | **-1.201** | **6.504** | -0.128 | 4.195 | 0.139 | 3.306 | 22.635 | 16.347 | 48.040 | 8.091 | -75.563 | 39.339 |
| Clay | General | | **1.667** | **6.205** | **1.318** | **3.934** | **0.893** | **3.147** | 10.910 | 17.353 | 2.549 | 18.120 | 53.308 | 2.800 |
|  | Site | IS | **1.393** | **6.662** | **0.674** | **4.166** | 1.197 | 3.178 | 1.746 | 19.467 | -2.090 | 20.901 | -6.175 | 5.434 |
|  |  | RS | **2.218** | **5.675** | **2.515** | **3.523** | 0.881 | 3.049 | 28.351 | 11.910 | 18.654 | 11.336 | 79.270 | 3.311 |
| PD | General | | **0.466** | **5.323** | **0.028** | **4.103** | -0.376 | 4.386 | 18.883 | -30.118 | 28.335 | -56.401 | -37.415 | 116.916 |
|  | Site | IS | **1.411** | **3.215** | **0.775** | **2.212** | 0.106 | 3.206 | 46.139 | -102.626 | 48.777 | -109.297 | -1.610 | 8.187 |
|  |  | RS | **-1.265** | **9.305** | **-1.460** | **7.810** | -1.590 | 7.303 | -21.453 | 71.930 | -19.387 | 61.558 | -43.151 | 139.742 |

*Note*: Designations: RS -reference sites, IS -impacted sites. Soil properties: P -phosphorus (mg/dm^3^), K -potassium (cmolc/dm^3^), Ca -calcium (cmolc/dm^3^), Mg -magnesium ( (cmolc/dm^3^), Al -aluminum ( (cmolc/dm^3^), C -carbon (g/kg), S -sulfur (mg/dm^3^), Cu -copper ((mg/dm^3^), Mn -manganese (mg/dm^3^), Fe -iron ((mg/dm^3^), Zn -zinc ((mg/dm^3^), Bsat -base saturation (%), Cs -coarse sand (kg/kg), Fs -fine sand (kg/kg), Silt (kg/kg), Clay (Kg/kg), PD - Particle density (g/cm^3^).
